# Functional Correlates of Positional and Gender-Specific Renal Asymmetry in *Drosophila*

**DOI:** 10.1371/journal.pone.0032577

**Published:** 2012-04-04

**Authors:** Venkateswara R. Chintapalli, Selim Terhzaz, Jing Wang, Mohammed Al Bratty, David G. Watson, Pawel Herzyk, Shireen A. Davies, Julian A. T. Dow

**Affiliations:** 1 Institute of Molecular, Cell and Systems Biology, College of Medical, Veterinary and Life Sciences, University of Glasgow, Glasgow, United Kingdom; 2 Strathclyde Institute for Pharmacy and Biomedical Sciences, Glasgow, United Kingdom; National Cancer Institute, United States of America

## Abstract

**Background:**

In humans and other animals, the internal organs are positioned asymmetrically in the body cavity, and disruption of this body plan can be fatal in humans. The mechanisms by which internal asymmetry are established are presently the subject of intense study; however, the *functional significance* of internal asymmetry (outside the brain) is largely unexplored. Is internal asymmetry functionally significant, or merely an expedient way of packing organs into a cavity?

**Methodology/Principal Findings:**

Like humans, *Drosophila* shows internal asymmetry, with the gut thrown into stereotyped folds. There is also renal asymmetry, with the rightmost pair of renal (Malpighian) tubules always ramifying anteriorly, and the leftmost pair always sitting posteriorly in the body cavity. Accordingly, transcriptomes of anterior-directed (right-side) and posterior-directed (left-side) Malpighian (renal) tubules were compared in both adult male and female *Drosophila*. Although genes encoding the basic functions of the tubules (transport, signalling) were uniformly expressed, some functions (like innate immunity) showed positional or gender differences in emphasis; others, like calcium handling or the generation of potentially toxic ammonia, were reserved for just the right-side or left-side tubules, respectively. These findings correlated with the distinct locations of each tubule pair within the body cavity. Well known developmental genes (like *dorsocross, dachshund* and *doublesex*) showed continuing, patterned expression in adult tubules, implying that somatic tissues maintain both left-right and gender identities throughout life. Gender asymmetry was also noted, both in defence and in male-specific expression of receptors for neuropeptide F and sex-peptide: NPF elevated calcium only in male tubules.

**Conclusions/Significance:**

Accordingly, the physical asymmetry of the tubules in the body cavity is directly adaptive. Now that the detailed machinery underlying internal asymmetry is starting to be delineated, our work invites the investigation, not just of tissues in isolation, but in the context of their unique physical locations and milieux.

## Introduction

**Figure 1:**
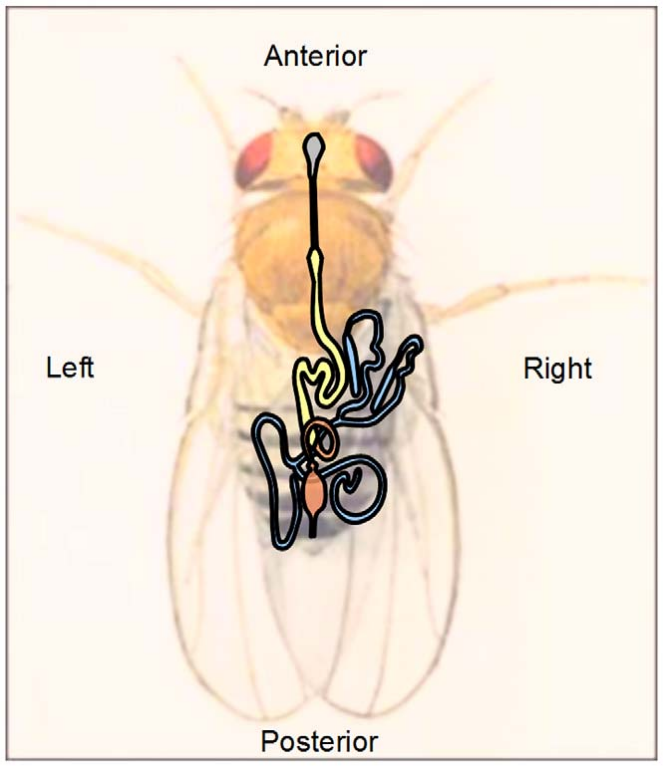
Internally asymmetric organisation of the *Drosophila* alimentary canal. The alimentary canal is divided into ectodermal foregut (grey), endodermal midgut (yellow) and ectodermal hindgut (orange). The midgut and hindgut are thrown into tightly defined loops and folds, the most obvious consequence being that the two pairs of Malpighian (renal) tubules (blue) are oppositely directed. The right pair (with conspicuous dilated initial segment) is always directed anteriorly and wraps around the midgut; whereas the left pair are directed posteriorly and associate with the hindgut.

The vertebrate body plan is internally asymmetric [1], [2], [3], and although complete *situs inversus* is benign in humans, partial disruption of this asymmetric organization (for example in heterotaxy) can be lethal [4], [5], [6]. Although separated by 450 M year of divergent evolution, many insect body plans show similar internal asymmetry; for example, the alimentary canal of the classical developmental model *Drosophila* starts development symmetrically, but is then thrown into highly stereotyped folds within the body cavity. Just as human kidneys sit slightly differently in the abdomen, so the *Drosophila* Malpighian (renal) tubules show marked asymmetry; the two tubule primordia initially sit dorsoventrally at the midgut / hindgut boundary, then a rotation of the developing alimentary canal sets the dorsal tubules on the right side, and the ventral tubules on the left side, of the body [7]. Remarkably the right-side tubules always elongate anteriorly within the body cavity into the thorax, while the left-hand tubules always grow posteriorly [8] (**Fig. 1**). There is a clear advantage to this distribution, as it ensures that the tubules ramify throughout the body cavity (haemocoel). Thus the haemolymph, although circulating only sluggishly, is nonetheless regulated efficiently.

However, the tubules also display morphological differences. The right-hand tubules contain conspicuous distal initial and transitional segments (**Fig. 1**). Although no such segments are visible in the left-hand tubules, genetic markers that label initial and transitional domains in the anterior tubules also label smaller and otherwise morphologically cryptic domains in the posterior tubule [9]. Unusually, the *Drosophila* Malpighian tubule is capable of providing both developmental and functional readouts [8], as its transport and signalling physiology has proved highly amenable to study [10]. Although the cells of the initial domain are thin and do not display prominent structural adaptations for ion transport (like abundant mitochondria or deep microvilli), this region is capable of excreting calcium at extremely high rates [11]. There is thus already some evidence of functional asymmetry in this simple kidney, and this is reinforced by asymmetric gene expression: the *homothorax*/*dorsotonals* transcription factor is expressed exclusively in the initial segment of the right-hand tubules [12].

The insect Malpighian tubule – and particularly that of *Drosophila* – is well understood at multiple levels [13]. It is a surprisingly good model for renal function and disease [14], including stem cell differentiation and cancer [15], [16], [17], [18]; and even the formation of kidney stones [19], [20]. The tubule, as a major osmoregulatory tissue with a wide range of transporters in common with mammalian kidney [12], [21], [22], [23], responds to desiccation stress, salt stress [24], [25], oxidative stress [26], [27], [28], immune stress [29], and plays a key role in the defence against xenobiotics [30], [31], [32], [33], [34]. It is also a model for neuroendocrine control [35], [36], [37]. The *Drosophila* Malpighian tubule thus offers a particularly useful, and broadly relevant, system in which to address issues of asymmetry.

One approach to address this and other possible asymmetries in the tubule is to compare the transcriptomes of right- and left-hand tubules; so in this study we performed a comprehensive analysis, using Affymetrix version 2 chips on left- and right-hand tubules, and further subdivided by sex. The results showed previously unsuspected lateral or sex-specific asymmetry in expression in a diverse set of genes, including those involved in positional specification in the embryo, and others involved in signalling. The data thus allow a reappraisal of functional sequelae of positional asymmetry in somatic tissues in higher organisms.

## Results

### Left v. Right: Genes Up-regulated in Right-side Tubules Suggest an Interplay with the Gut

**Figure 2:**
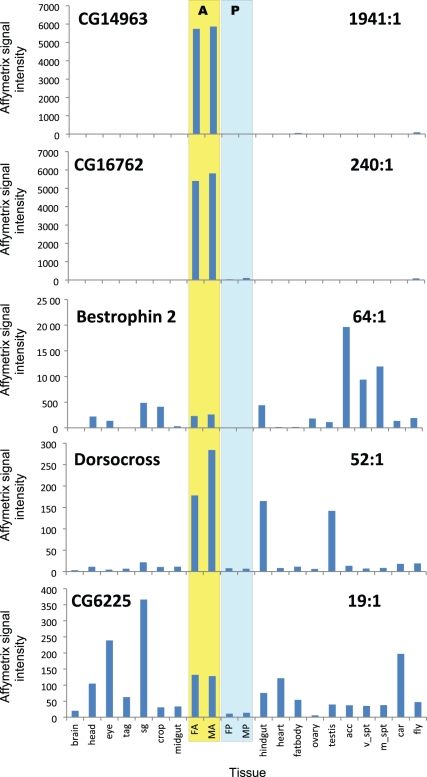
Adult tissue expression profiles of the 5 genes with most enriched expression in anterior (right-side tubules). Y-axis shows mean Affymetrix microarray signal for each tissue along the x axis (N = 4 arrays). The data for anterior and posterior tubules are highlit and labelled A and P, respectively. Abbreviations: Tag - thoracicoabdominal ganglion; sg - salivary gland; FA, MA, FP, MP - female or male, anterior or posterior tubules; acc- male accessory glands; v_spt, m_spt – virgin or mated spermatheca; car – carcass; fly – whole fly. Data for tubules are from this paper: comparison data for other tissues is derived from FlyAtlas.org. For simplicity, error bars are omitted; these can be obtained by interrogation of FlyAtlas.org, but are typically in the range 5–10% of the signal.

As the posterior tubule appears morphologically similar to the anterior (right) tubule, but with a reduced initial segment [9], we expected to find the left tubule transcriptome to be broadly a subset of the right tubule. Indeed, of 88 genes significantly changed (at FDR<0.05), 74 genes were significantly up-regulated in right tubules, compared with only 14 in left tubules (**Table 1** **& Supporting information S1**). The results implicate the right-side tubules in defence and calcium handling roles; the 5 genes with most asymmetric expression are presented, together with their FlyAtlas-derived expression in other tissues, in **Fig. 2** **.**

*CG14963* is 1940x up-regulated in right (anterior) tubules, and FlyAtlas.org [38] reports that expression of this gene is utterly specific to the tubule. What function could be performed entirely by just two out of 4 tubules? *CG14963* encodes a protein containing an insect allergen related repeat, associated with nitrile-specific detoxification. To discourage herbivores, some plants (notably brassicas) have developed a glucosinolate-myrosinase chemical defence mechanism; and consequently insects, like caterpillars of the small white butterfly (*Pieris rapae*), have adapted to produce a detoxifying molecule, nitrile-specifier protein (NSP) [39]. BLASTP searches reveal that proteins closely similar to *CG14963* are found throughout the Diptera, but that the Dipteran branch is rather diverged from the Lepidopteran branch. The implication is that *CG14963* is part of a Dipteran adaptation to dietary challenge. Why then is this gene confined to, and expressed at high level in, the right-hand tubules (Fig. 2)? The key observation is that the right-hand tubules ramify anteriorly to surround the midgut, whereas the left-hand tubules are confined to the posterior abdomen, where they surround the hindgut. The tubules are major tissues of detoxification [31]; so if a toxic compound were released by digestion, and passed through the highly permeable midgut, it would thus be a task for primarily the anterior tubules to neutralize the compound. Conversely, the left tubules might be expected to be enriched for genes involved in detoxifying compounds leaching from the hindgut; this will be demonstrated later.

**Table 1:** The 10 most anterior, posterior, female and male-enriched genes.

Probeset ID	Gene Title	*P*-value	Fold-Change
**Anterior-enriched**			
1638974_at	*CG14963*	1.9E-19	1940.9
1640881_at	*CG16762*	1.7E-10	239.7
1634031_at	*Bestrophin 2*	1.4E-10	63.6
1631218_at	*Dorsocross*	5.0E-12	52.4
1627384_at	*CG6225*	2.8E-08	18.5
1629459_at	*Dorsocross3*	3.8E-12	16.7
1635591_at	*ninjurin A*	2.7E-10	15.2
1625266_at	*antigen 5-related*	1.1E-05	12.5
1628302_at	*CG13748*	1.4E-09	10.6
1635398_at	*CG10587*	2.8E-08	6.2
**Posterior-enriched**			
1624060_at	*bric-a-brac*	1.7E-04	–1.5
1626439_at	*CG15353*	1.8E-04	–1.9
1635803_s_at	*bowel*	8.7E-07	–1.9
1634418_at	*CG33281*	1.5E-07	–2.4
1637123_a_at	*CG33281*	3.5E-07	–2.7
1627354_at	*CG11779*	1.3E-04	–3.3
1634658_a_at	*CG42708*	2.0E-05	–3.8
1625629_at	*Esterase-6*	3.9E-08	–3.8
1639637_a_at	*CG3376*	5.1E-07	–7.6
1629738_at	*CG14957*	9.2E-07	–9.0
**Female-enriched**			
1629545_at	*yolk protein 1*	5.4E-07	515.7
1631419_at	*yolk protein*	4.4E-07	514.0
1623655_at	*yolk protein 2*	2.3E-07	387.7
1633540_at	*CG8147*	5.0E-07	39.9
1630600_at	*Frost*	2.1E-05	31.4
1637702_at	*---*	1.2E-07	28.8
1641419_at	*attacin*	6.7E-04	25.1
1633820_at	*Fad2*	3.5E-04	22.5
1623776_s_at	*doublesex*	1.4E-10	19.3
1627613_at	*Metchnikowin*	7.1E-05	12.0
**Male-enriched**			
1635189_at	*drosomycin*	1.9E-03	–6.8
1638816_at	*CG3884*	1.1E-03	–6.8
1628982_at	*neuropeptide F receptor*	7.0E-07	–6.9
1632636_at	*CG2145*	3.1E-09	–8.6
1638484_at	*Gene 3*	2.7E-04	–10.6
1640799_at	*doublesex*	6.9E-13	–10.8
1628611_at	*CG11241*	3.5E-09	–11.1
1637145_at	*CG14787*	6.4E-10	–18.3
1633432_at	*CG9657*	4.9E-12	–41.6
1633275_at	*CG31562*	7.8E-10	–56.8

Complete lists are in Supporting information S1.

A similar defensive role is implied for *CG16762*, another gene strongly (240x) up-regulated in right-hand tubules (**Fig. 2**), and with tubule-restricted expression. BLASTP searching reveals similarity to TsetseEP, a mucin implicated in tsetse susceptibility to trypanosome attack. As all BLASTP hits are confined to the Schizophora, the gene family may represent a highly specific adaptive radiation within this subset of the Diptera, to defend against microbial attack. Although Drosophilids are not susceptible to trypanosome attack, dog heartworm (*Dirofilaria immitis*) is an intracellular parasite of the Malpighian tubules of culicid mosquitos; so there may be a general requirement for the tubule to defend against luminal pathogens.

**Figure 3:**
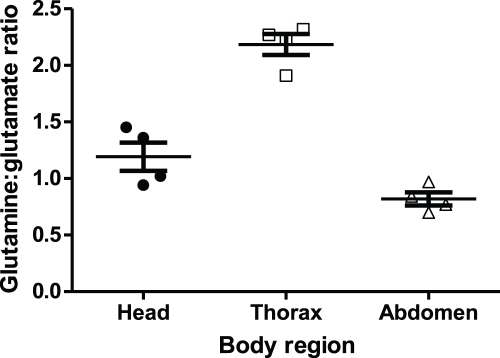
Glutamine: glutamate ratios in acutely dissected head, thorax and abdomen of adult flies. Box plots denote median and interquartile ranges, with individual replicates (4 per tissue), marked.

Conspicuously enriched (3.8x) in the left-side tubules, which wrap around the hindgut, is probe set 1634658_a_at (**Table 1**), which corresponds to *CG42708*, a mitochondrial glutaminase [40]. FlyAtlas reports that this gene is particularly highly expressed in the tubules and hindgut. Glutaminase generates ammonia stoichometrically in the generation of glutamate from glutamine, and is found in kidney and liver in mammals: in kidney, the resulting ammonium ion can be used to rescue bicarbonate in the collecting duct [41]. Although abundantly produced in freshwater insects (where it can effectively diffuse away to harmless levels), ammonia is generally held to be a toxic and undesirable metabolite in terrestrial insects [42]. Our data suggest that this reaction is concentrated in tissues in the back end of *Drosophila*, thus compartmentalizing it away from other potentially sensitive tissues. To confirm the spatial segregation of glutaminase activity, we measured the glutamine: glutamate ratio in acutely-dissected head, thorax and abdomen in adult flies, showing that the ratio is lowest in the abdomen (**Fig. 3**).

**Figure 4:**
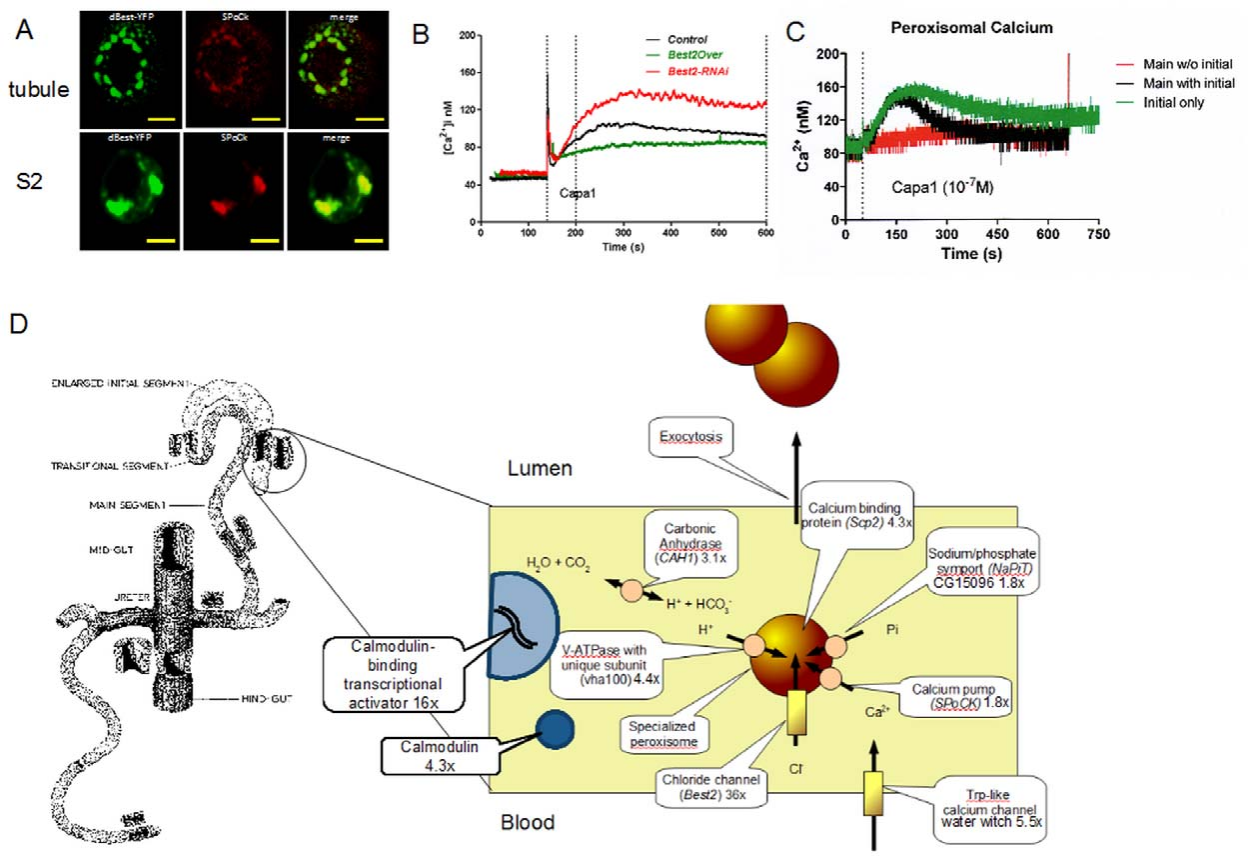
Asymmetric calcium excretion deduced from anterior-specific gene expression. (**A**) **Best2 colocalizes with peroxisomal SPoCk-C.** Heterologous and ectopic expression of Best2-YFP and its colocalisation with native peroxisomal localized secretory pathway Ca^2+^/Mn^2+^ ATPase isoform C (SPoCk-C). The first panel of images shows the *in vitro* colocalisation (in S2 cells) of heterologously expressed yellow fluorescent protein fused Best2 (Best2-YFP) with native SPoCk-C (using affinity-purified rabbit anti-SPoCk-C antibody at 1:1000 [51]) and the second panel represents the same but *in vivo* colocalisation in adult tubules. Scale bars denote 10 µm. (**B**) **Calcium changes in initial segment depend on Best2 expression.** Real-time calcium traces in tubule initial segment principal cells, in response to the calcium-mobilizing diuretic peptide capa1, measured with targeted transgenic aequorin. Both over-expression and knockdown of *Best2* modulates the cellular calcium signal. (**C**) **The major peroxisomal calcium pool is in initial segment.** Peroxisomal calcium traces in response to capa1 from tubules, tubules without initial segments, or microdissected initial segments alone. (**D**) **Schematic enlargement of the initial segment of the anterior Malpighian tubule,** outlining processes thought to underlie the formation and secretion of calcium-rich ‘spherites’, and labelled with some anterior-enriched candidate genes.

*Bestrophin 2* encodes (of four *Drosophila* bestrophins) the most closely related to human bestrophin 1, a calcium- and volume-activated chloride channel of retinal pigmented epithelial cells, which is mutated in Best’s macular dystrophy [43], [44]. However, Bestrophins play wider roles: they are implicated in epithelial-to-mesenchymal transitions in human kidney [45], and in olfactory transduction [46]. In *Drosophila*, *Best1* also mediates a calcium and volume activated chloride current [47]. FlyAtlas reports that, although expressed in eye, *Best2* is abundant in epithelia; in adult tubule, it is 64x enriched in right-hand tubules (**Fig. 2**). The tubules secrete fluid at high rates, and chloride conductance is under control of the diuretic neuropeptide leucokinin, which acts through intracellular calcium [48], [49]. However, Best2 is unlikely to be the major chloride channel of the tubule, because it is confined to the right-hand tubules, whereas leucokinin acts equally on both right and left tubules. An alternative is that Best2 mediates selective loading of specialized peroxisomes with calcium concretions; the initial segments of the right-side tubules are known to excrete calcium at remarkable rates [11], forming calcium rich concretions [50], derived from specialized peroxisomes containing an isoform of the SPCA Ca^2+^ ATPase [51]. Best2 also localizes to peroxisomes, both *in vivo* and in S2 cells *in vitro* (**Fig. 4A** **, Supporting information S1**). Manipulation of *Best2* expression levels impacts on cellular calcium, consistent with a key role for this gene in calcium handling in the initial segment (**Fig. 4B**).

### A Mechanism for Asymmetric Calcium Handling

The only known functional asymmetry in tubule function is the anterior-specific storage excretion of calcium as phosphate-rich mineral concretions in the enlarged initial segments of the right-side tubules [11]. Based on results implicating SPoCk, the secretory pathway Ca^2+^/Mn^2+^ ATPase, [51] and Best2 (**Fig. 4A-B**) in calcium handling in tubule initial segment, it is possible to build a model for spherite formation in the tubule. Physiological data had implicated the initial segments as particular hot-spots for calcium transport [11]; and our data placing a peroxisome-targeted isoform of SPoCk on initial segment vesicles, was confirmed by direct recording of peroxisomal calcium, showing that the major peroxisomal calcium pool in tubules was in initial segments (**Fig. 4C**). Interestingly, the right-side tubule transcriptome is enriched for genes implicated in calcium and phosphate transport, and for peroxisomal biogenesis, allowing a conceptual model for spherite generation to be built (**Fig. 4D**). Calcium could be admitted to the specialized peroxisome by entry through a trp-like plasma membrane channel, together with a peroxisomal isoform of SPoCk; phosphate is provided by the Na^+^/phosphate cotransporter, NaPiT; the membrane is polarized by a specialized V-ATPase (with protons provided by a highly enriched isoform of carbonic anhydrase), and charge balance provided by the Best2 chloride channel. Calcium is stabilized in the peroxisome by calexcitin, a specialized calcium-binding protein originally identified in sarcoplasmic reticulum. There is also scope for regulation of the pathway, because of high enrichment of calmodulin and a calmodulin-responsive transcriptional activator.

### Dorsocross Directs Laterally Asymmetric Expression

Given that there are real functional differences between left and right-side tubules, and that these are underpinned by significant transcriptional differences, it is interesting to start to identify the transcriptional programme involved in specifying asymmetry. The three *Dorsocross* transcription factors are found in a cluster on chromosome 3L. These T-box, p53-like, *brachyury* transcription factors were originally implicated in heart formation [52] at the dorsal midline, under control of decapentaplegic and wingless. They are possibly the products of recent gene duplication, because they have similar embryonic expression profiles, and are partially redundant [52]. Post-embryonically, however, FlyAtlas [38] reveals differences in expression. *Doc1* is expressed mainly in tubule, hindgut and testis; *doc2* is confined to the hindgut, and *doc3* to tubule, hindgut and heart. Our data are consistent with reported embryonic expression in only the anterior tubules [52]; overexpression of *Doc* genes in the embryonic hindgut results in failure of the posterior tubules to develop, leaving only the anterior pair [53]. Although *Doc* genes are implicated in the very earliest stages of tubule specification, these data show that they continue to be expressed in the same spatially restricted pattern (*Doc1* is 52x, and *Doc3* is 17x, enriched in anterior rather than posterior tubule) through larval and into adult life, suggesting that the anterior/posterior distinction remains functionally significant throughout life, and that continued expression of *Doc* genes (or *dorsocross* transcriptional network) likely maintains that identity.

**Figure 5:**
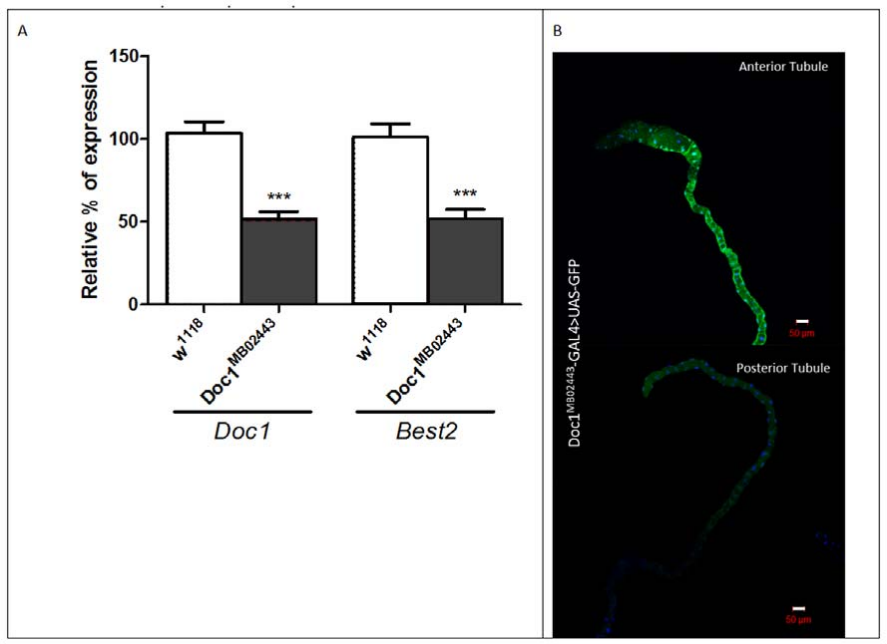
Asymmetric expression of *Best2* is under the control of the *dorsocross transcriptional network*. (A) *Doc1* and *Best2* expression in the anterior tubule are both reduced to 50% of wild-type levels in heterozygotes of the Mi{ET1} enhancer trap GAL4 insertional mutant, *Doc1^MB02443^*, in comparison to wild-type, *w^1118^*, flies. (B) *Doc1^MB02443^* driven GFP expression confirms *Doc1* anterior-enriched expression found in the microarrays and qRT-PCR. Scale bars denote 50 µm.

To test whether *Doc* genes indeed impact on the functional asymmetry of adult tubules, we examined expression of a right-side specific gene, *Best2*, in a *Doc1* hypomorph. In a *Doc1* heterozygote insertional mutant (with 50%-less *Doc1* expression than wild-type), *Best2* expression was significantly reduced (to 50% of wild-type levels; **Fig. 5A**). The Minos {ET1}[11] transposon [54] insertion in *Doc1* also drove GFP expression in anterior, but not posterior tubules (**Fig. 5B**), confirming that asymmetric expression of *Doc1* directly controls asymmetric expression of *Best2*.

Other transcription factors enriched in right-side tubules are *dachshund* (4.0x), the *ski* proto-oncogene homologue important in development of the legs and eyes, but not previously implicated in tubule function; and *homothorax*/*dorsotonals* (3.3x), also important in eye development. By contrast, *bric-a-brac* (1.5x), *bowl* (1.9x) and *suppressor of hairy wing* (1.2x) are all significantly enriched in the left-side (posterior) tubules (**Supporting information S1**).

### Gender Differences in Tubule Function

Male and female tubules show markedly distinct transcriptional profiles. Although most of the classical roles of the renal system (pumps and channels underlying the generation of a primary urine, and organic solute transporters for excretion of undesirable solutes) are expressed roughly equally in both sexes, 113 genes are up-regulated (≥2-fold) in female, and 116 genes in male, tubules (at FDR<0.05) (**Supporting information S1**). Both by inspection, and using the Gorilla gene ontology package [55], we identified several classes of genes with clearly sexually-dimorphic expression, particularly in lipid metabolism, sex determination, neuropeptide signalling and immunity/defence.

The yolk proteins, Yp1-3, encode lipases. Although rather broadly expressed (FlyAtlas.org), their expression is utterly sexually dimorphic, with F:M fold changes in excess of 380x. Other genes with enriched female expression implicated in fat or glyceride metabolism are *Fad2* (22x), a fatty acid desaturase; *CG3599* (6.5x), a biotinidase; and *imaginal disc growth factor 3* (5.9x), a glycoside hydrolase. Although the function of *female-specific independent of transformer* (*fit*- enriched 9.9x) is not known, it is expressed in fat cells in the head [56], and so *fit* may also impact on fat metabolism. The clear implication is that the maternal investment in yolk impacts on other tissues, and that enhanced lipid metabolism is a critical task in females.

### Sex Determination Genes and Sexual Dimorphism in Neuroendocrine Control

Understanding sex determination is a triumph of *Drosophila* developmental biology; however, there has been relatively little attention paid to the mechanism or physiological significance of maintenance of gender differences in post-embryonic somatic tissues. The expression data (**Supporting information S1**) clearly show that some of the best-known players in embryonic sex determination persist for the lifetime of the insect, presumably to maintain sexually dimorphic gene expression. For example, *doublesex* appears in both male-enriched (10x) and female-enriched (19x) lists, because there are distinct probe sets for the male and female transcripts of this gene. *In situ* hybridization confirms the enriched expression of *Doublesex-RA* in male tubules compared to female tubules. Similarly, *Sex-lethal* is enriched (2.8x) in male tubule, as is the dosage-compensation gene, *male-specific lethal*, *msl-2* (3.4x). It is thus clear that adult tubules maintain a clear gender identity in terms of control of gene expression. Given the differing metabolic loads required for reproduction, and the implicit requirements for absorption, excretion and osmoregulation, it is reasonable that the tissues in the alimentary canal possess robust and lifelong gender identity. Although the popular view is that gender is important in development and the brain, the very clear message is that somatic tissues care about their sex too [57].

If the tubules exhibit sexually dimorphic expression patterns, it is reasonable to expect that their endocrine control mechanisms might also diverge. Intriguingly, two well-known receptors (for sex peptide and neuropeptide F) are prominently male-enriched (**Supporting information S1**). Their spatial expression patterns are presented along with their peptide ligands in **Supporting information S1**. *Sex peptide receptor* (*SPR*) is 6.6x male-enriched. Sex peptide is transferred with the sperm to the female, where it induces a series of changes in physiology and behaviour, notably a reduction in receptivity to subsequent matings [58]. Not surprisingly, therefore, FlyAtlas reports high levels of the receptor in (female) spermathecae, the receptacles for sperm after mating. Selective expression of a neuropeptide targeted at females in male tubules is thus puzzling, but could be explained by the recent observation that myoinhibitory peptides (MIPs) are the ancestral ligands for SPR, as SPR homologues are found even in insects without an obvious sex peptide [59]. Furthermore, *Drosophila* SPR is 10x more sensitive to the *Drosophila* MIP, allatostatin-B [60], [61] than to SP itself [59]. The data may thus have uncovered a previously unknown neuropeptide control mechanism for the tubule; allatostatin B-reactive cells are found at the junction between the anterior and middle midgut [62], offering a direct signalling interaction between the gut and the tubule.

A second surprise is the male-specific expression of *neuropeptide F receptor 1* (*NPFR1*) [63], [64], [65], [66], [67], *Drosophila* NPF appears to be a homologue of vertebrate Neuropeptide Y. Although sNPF is only distantly related to NPF, and the two peptides are not redundant, they still perform similar roles [67]. Neuropeptide F/Y signalling regulates physiological processes including feeding behaviour, metabolism, reproduction and stress responses both in vertebrates and invertebrates, and there is an extensive literature implicating neuropeptide F signalling in clock function, learning, appetite and growth in *Drosophila* [68], [69]. However, FlyAtlas reveals that *NPFR1* is conspicuously enriched in the adult tubule; and its expression in tubules is 6.9x male-enriched (Supporting information S1). This was confirmed by qPCR (6.3±0.96x (*N* = 3) male enriched), suggesting that male tubule could be a major target for NPF. This nicely links with the observation that expression of NPF itself in the brain is sexually dimorphic, with expression in some neurons being male-specific and under the control of transformer and fruitless [70]. By qPCR, we found that NPF expression is also male-enriched in both head and body (**Supporting information S1**). FlyAtlas expression profiles are also presented for reference in **Supporting information S1**. Thus both the peptide and its receptor show sex-specificity.

**Figure 6:**
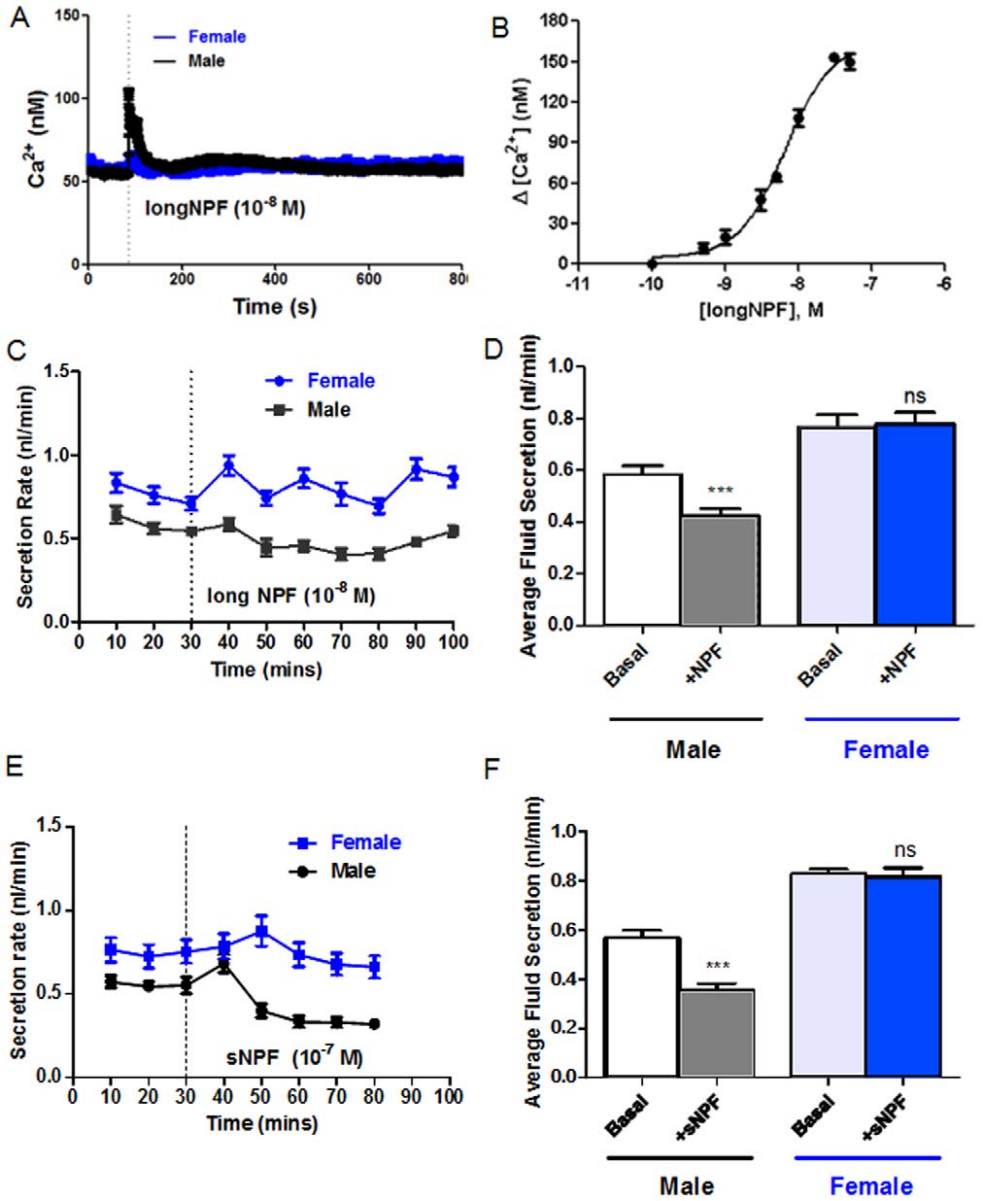
Male-specific signalling by Neuropeptide F and short neuropeptide F. (A) Typical traces showing differential real-time cytosolic calcium responses of male and female tubules. NPF was added to 10^–8^ M final concentration at 100 s. (B) Dose-response curve for NFP-induced calcium signalling in male tubules. (C) Typical trace showing response of tubules to NPF, added to 10^–8^ M final concentration at 30 min. Data are shown as mean±SEM (*N* = 10 tubules). (D) NPF inhibits fluid secretion in male, but not female, tubules. Average fluid secretion rates are compared before and after addition of 10^−8^ M NPF. *** denotes significant reduction, Student’s *t*-test, two tailed, P<0.05. (E) Typical trace showing response of tubules to sNPF, added to 10^−7^ M final concentration at 30 min. Data are shown as mean±SEM (*N* = 10 tubules). (F) sNPF inhibits fluid secretion in male, but not female, tubules. Average fluid secretion rates are compared before and after addition of 10^−7^ M sNPF. *** denotes significant reduction, Student’s *t*-test, two tailed, P<0.05).

What is the likely output of the signalling pathway? In heterologous systems, *Drosophila* NPFR signals through G*i* to inhibit adenylate cyclase [64]. As the tubules contain cyclic-nucleotide gated calcium channels [71], [72], measurement of intracellular calcium provides a useful surrogate assay for NPF function. Consistent with the microarray results, NPF transiently elevates [Ca^2+^]*_i_* (**Fig.** **6A**) in a dose-dependent manner (**Fig. 6B**) in male, but not female tubules, with an EC_50_ of 6 nM (**Fig. 6B**), confirming that the tubules receive a *bona fide* NPF signal through NPFR1. Consistent with a likely action through G*_i_*, resting fluid secretion was decreased, but only in males (**Figs 6C-D**).

A distantly related peptide, the so called short neuropeptide F (sNPF) has similar, but independent actions on feeding [66], [67], [68], and the cognate receptor (*CG7395*/*sNPF-R*) is also selectively enriched (1.4x) in male tubules; so the impact of sNPF on tubule function was also assessed. Again, sNPF inhibits resting fluid secretion, but only of male tubules (**Figs 6E-F**). These results thus both identify two new agonists acting on the tubule, and emphasise male-specific somatic roles for these two well-studied peptides.

An interesting possibility is that NPF may act locally to signal particular stress conditions: disruption of the NPF/NPFR1 circuit renders flies resistant to ethanol intoxication [73]; and the tubule is known to be a major site of expression of detoxification genes [31], such as alcohol dehydrogenase [12]. The tubule also integrates response to diverse stressors, such as salt stress [24] and immune challenge [25], [29], and so NPF may be modulating such responses. Again, NPF-reactive cells, which co-express the diuretic neuropeptide leucokinin, are found in the anterior midgut [62] as well as the CNS, suggesting that the tubule could receive direct warning of dietary stressors as they arrive in the midgut [74], together with a known diuretic signal. *Drosophila* tachykinin (DTK) peptide, short neuropeptide F (sNPF) and ion transport peptide (ITP) precursors have recently been shown to be colocalized in five pairs of large protocerebral neurosecretory cells in two clusters (designated ipc-1 and ipc-2a) in the adult flies [75]. The same report proposed that IPTs may act as antidiuretic hormones that regulate transport in hindgut. In addition to neuronal functions, sNPF has been implicated in regulation of insulin signaling, feeding and growth [67]. Thus our results support a complex network of central and peripheral signalling of NPF and sNPF in the modulation of clock-controlled functions, insulin signaling, feeding and growth. Immunity and detoxification.

The Malpighian tubule is a major immune [29], [76] and detoxification tissue, and express a cluster of immune/defence genes, which FlyAtlas suggests are expressed in broadly similar patterns - typically in the major immune tissues (head, midgut, tubule, fat body and spermathecae). However, these results indicate that there is gender-specific expression of several immune genes in tubule, suggesting males and females meet differing immune challenges. Female-enriched genes include the antimicrobial peptides *attacin* (25.1x), *Metchnikowin* (12x) and *immune induced molecule 2* (7.4x); the *peptidoglycan receptor protein SB1* (9.3x), an *N*-acetylmuramoyl L-alanine amidase with bactericidal properties against *Bacillus* spp. [77]; and the detoxification enzyme, cytochrome P450 *cyp4e3* (6.5x). By contrast, males preferentially express *drosomycin* (7.8x), and a series of cytochrome P450s, including the cardinal insecticide resistance locus, *cyp6g1* (2.5x) [31].

This enrichment for immune genes suggests that although females and males make similar overall investments in innate immune response, females experience distinct immune challenges from males, which are argued to emphasise defence against Gram-negative bacteria more than females [78].

## Discussion

The key outputs of this work are: detailed transcriptomic datasets that outline the scope and extent of differences between male and female, and left and right insect Malpighian tubules; interesting novel hypothesis on the spatial and gender compartmentalization of different processes; and preliminary experimental data that suggest that some of these transcriptomic differences really do have physiological significance.

For the first time, a transporting epithelium has been profiled for expression according to both its gender and its disposition within the body. The results show that all tubules are not equivalent; although they perform the same key functions (the generation of urine, detoxification and excretion of waste material) they also perform some tasks (like innate immune defence) with different emphases; and some tasks, (like calcium homeostasis) are remarkably dimorphic in their transcriptional repertoire. The neuroendocrine control of tubules, though substantially the same for all, also shows subtle distinctions. Importantly, it appears that the same transcription factors which determine positional identity and gender in the early embryo persist into adulthood, implying that identity of the tubules remains important, and that these transcription factors must be considered to be important for more than early development.

**Figure 7:**
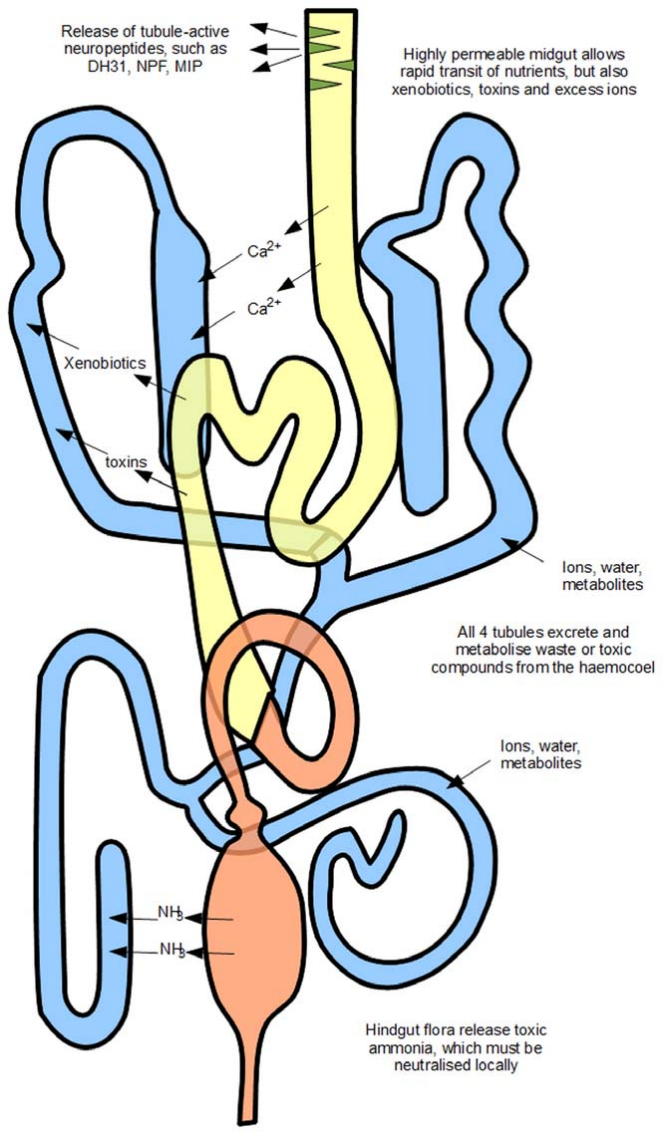
Model for universal and spatially asymmetric functions of the renal system. The fundamentals of the renal system, to generate urine and to transport and metabolize metabolites and toxins, are common to both sets of tubules. However, the right-side tubules ramify anteriorly and associate intimately with the highly permeable midgut. They need to play a particular role in transport or sequestration of solutes arriving from the gut. The conspicuous adaptation is for the storage excretion of calcium. The left-hand tubules perform a corresponding task for the hindgut, to which they are loosely attached. Here, the major pressure is to guard against excessive release of ammonia from nitrogenous excretion.

In this work, we found that morphological asymmetry has direct relevance to functional optimization of complex tissues in the whole organism, specifically putting transport processes near the organs that generate their substrates, and with both local and central control of output. A summary diagram is shown in **Fig. 7**. In this model, the basic fluid transport and homeostatic functions of the tubule are common to left and right tubules; but functional sequelae result from the proximity of the right-side tubules with the highly permeable midgut, and the left-side tubules with the excretory and concentrative power of the hindgut. Both dietary toxins and overabundant solutes (like calcium) must be sequestered rapidly by the tubules before they impact on the whole insect. Local signalling by neuroendocrine cells embedded along the length of the midgut [62] could complement central signals, by relaying information about the gut contents directly to the tubules. The compartmentalization of ammonia handling to the hindgut and closely-coupled left-side tubules would equally protect the insect against toxic levels of an essential metabolite.

We and others have previously argued that the high number of anonymous (undocumented) genes with highly tissue-specific expression patterns found in FlyAtlas [38] suggests that researchers need to become more interested in specific tissues within the organism, in order to functionally characterize the whole genome. This principle is demonstrated further here; although we have previously-identified novel tubule-specific genes [12], the present dataset shows that the principle further extends to sidedness and to gender, offering fascinating insights as to likely novel functions. The power of *Drosophila* genetics will allow a detailed examination of the interactions between tissues in their natural physical layout within the whole organism, and so allow an understanding of gene functions in an organotypic, organismal context. In an insect, this level of understanding is of great benefit to developing insights that might lead to novel control methods; but it further suggests that there may be asymmetry to be uncovered in other epithelia in the future.

## Methods

### Drosophila

*Drosophila melanogaster* (Canton S, wild type) were reared on standard diet at 25°C and 55% relative humidity on a 12∶12 h photoperiod. Malpighian tubules were dissected from 7–10 day old adults, for compatibility with the extensive physiological literature on the tubule [48], [79], [80], [81], [82], [83], [84], [85]. At this stage, the tubules are in a relatively stable state after adult emergence, and their secretion parameters do not change detectably between 3 and 14 days post-emergence.

### Microarrays

Tubules were quickly dissected in cold Schneider’s medium (Invitrogen, UK) and collected into RNA stabilization buffer RLT (Qiagen, UK) every 5 min to ensure the integrity of the mRNA. Care was taken to sever the tubules from the gut at the junction of lower ureter and hindgut (**Fig. 1**) so that no other tissue was included in the sample. Total RNA was extracted from wild type Canton S flies using RNeasy columns (Qiagen, UK). RNA was quantified with NanoDrop-1000 (NanoDrop Technologies, USA), and quality assessed using Bioanalyzer (Agilent Technologies UK Ltd.). For each sample, 4 biological replicates, each consisting of 40 tubules, were generated. Samples of 100ng of total RNA were reverse-transcribed, then *in vitro* transcribed, according to Affymetrix standard 2-cycle small sample protocol. The quality of the complementary RNA (cRNA) was also checked on the Bioanalyzer, with a sample in which the broad cRNA peak exceeded the height of the low molecular weight degradation peak taken to be satisfactory. Samples were then run on the Affymetrix *Drosophila* genome chip v.2, comprising 18880 probe sets analysing over 18500 transcripts, under the Manufacturer’s standard conditions.

### Microarray Data Analysis

Microarray data analysis and quality control were performed initially using Affymetrix GCOS software, and with the Partek Genome analysis suite. A 2-way ANOVA approach was employed to identify genes that were differentially expressed by gender and by tubule position (left or right). We report genes with significant differential expression, based on a false discovery rate (FDR) threshold of *P* = 0.05. The microarray data have been deposited at NCBI GEO, with the accession number (GSE7763).

Although our previous study had shown a close correlation between Affymetrix microarray and quantitative real-time PCR (qPCR)-derived measures of expression level within a tissue [12], a selection of genes was further validated by qPCR and *in situ* hybridization (**Supporting information S1**).

### qRT-PCR

Samples were prepared as described above and qRT-PCR was performed using Opticon DNA engine 4 (Bio-Rad Technologies UK) using Sybr green master mix (Finnzymes, UK) with the primer pairs described in **Supporting information S1**. The data obtained was then expressed as fold change on the basis of C_T_ values. Student’s *t* test (unpaired; 95% confidence interval) was performed with GraphPad Prism statistics software (GraphPad Software Inc., USA).

### In situ Hybridization

DIG-labelled mRNA probes were transcribed from PCR products, generated using the same primers (**Supporting information S1**) used for qRT-PCR. Adult Malpighian tubules were dissected in Schneider’s medium (Invitrogen, UK) and placed into wells of a Millipore 96-well plate (MAGVN22 or MAGVS22) with 100 µl of Schneider’s medium. The *in situ* protocol is adopted from the Berkeley *Drosophila* Genome Project (BDGP) (http://www.fruitfly.org/about/methods/RNAinsitu.html).

### Measurement of Glutamine : Glutamate Ratios

Acutely dissected heads, thoraces and abdomens from ten 7-day old adults (mixed sexes) were homogenised in ice cold methanol/chloroform/water (3∶1∶1), followed by sonication for 15 s. The supernatant was then centrifuged for 5 minutes at 13000 *g* at 0°C, and the filtered supernatants were then stored in a freezer at −80°C until required. The samples were then analysed using ZICHILIC chromatography interfaced to an LTQ Orbitrap mass-spectrometry as described previously [20], [86]. Four independent samples were analysed for each tissue. Data were plotted as means and interquartile ranges, and difference between groups tested with the Kruskal Wallis nonparametric test.

### Generation of Peroxisomal Calcium Reporter Transgenic Flies

*Drosophila* transgenic for a luminescent peroxisomal-targeted calcium reporter were generated by cloning a peroxisomal-targeted *Aequorea victoria* apoaequorin construct into the pUAST vector. This construct uses the KVK-SKL amino acid motifs, previously shown to target YFP to peroxisomes [87]. The sequence coding for the KVK-SKL was introduced before the stop codon of the aequorin template by PCR using the oligonucleotides 5′-GCAGATCTATGACAAGCAAACAATACTCA-3′ and 5′-GCTCTAGATTACAGCTTGGACTTCACCTTGGGGACAGCTCCACC-3′. The resulting PCR product was digested with BglII and XbaI and ligated into the pUAST vector. Transgenic lines were generated using standard methods for P-element-mediated germline transformation (BestGene Inc, USA).

### Calcium Measurement

Cytosolic [Ca^2+^]_cyto_ or peroxisomal [Ca^2+^]_perox_ calcium was measured in tubules expressing transgenic aequorin according to published protocols [88], [89]. Briefly, the tubules were dissected, incubated at room temperature with 2.5 µM coelenterazine to reconstitute aequorin for 3 h. Calcium-dependent aequorin luminescence was measured in 10 biological replicates each containing 20-pairs of tubules. At the end of each experiment, total aequorin was released using 300 µl of lysis buffer (1% (v/v) Triton X-100, 100 mM CaCl_2_) in order to calculate the total luminescence; and the data were back-integrated using a program written in Perl, based on the method described by Button and Eidsath [90]. Data are presented as the mean (±SEM) and statistical significance was calculated using unpaired *t*-test.

### Tubule Fluid Secretion Assay

Classical fluid secretion assays were performed as previously described by Dow et al. [91]. Briefly, basal fluid secretion rates of the dissected live tubules were established for 30 min after which the required concentrations of peptides were added and secretion was measured for a further 70 min. The rates of secretion (in nl/min) were represented as the mean (±SEM) of approximately 30–35 tubules coming from 3 independent biological replicates.

### Pharmacology

sNPF (AQRSPSLRLRF-NH_2_) and MIP (AWQSLQSSW-NH_2_) peptides were synthesised by Biomatik, USA with a purity of greater than 98%. NPF (SNSRPPRKNDVNTMADAYKFLQDLDTYYGDRARVRF-NH_2_) peptide was a kind gift from Professor Ian Orchard (University of Toronto Mississauga). According to the peptide profiles generated by peptide profile calculator (Gene Script, UK), they were dissolved first in water to produce a stock solution of 10 mM and subsequently diluted in the Schneider’s medium to get required final concentrations.

## Supporting Information

Supporting information S1 **The combined supplementary information file comprises Figures S1 to S4 and Tables S1 to S4.**
(DOCX)

## References

[1] Burn SF, Hill RE (2009). Left-right asymmetry in gut development: what happens next?. Bioessays.

[2] Levin M, Palmer AR (2007). Left-right patterning from the inside out: widespread evidence for intracellular control.. Bioessays.

[3] Lopez-Gracia ML, Ros MA (2007). Left-right asymmetry in vertebrate development..

[4] Kartagener M, Stucki P (1962). Bronchiectasis with situs inversus.. Arch Pediat.

[5] Sharma N, Berbari NF, Yoder BK (2008). Ciliary dysfunction in developmental abnormalities and diseases.. Curr Top Dev Biol.

[6] Sutherland MJ, Ware SM (2009). Disorders of left-right asymmetry: heterotaxy and situs inversus.. Am J Med Genet C Semin Med Genet.

[7] Coutelis JB, Petzoldt AG, Spéder P, Suzanne M, Noselli S (2008). Left-right asymmetry in Drosophila.. Semin Cell Dev Biol.

[8] Beyenbach KW, Skaer H, Dow JAT (2010). The developmental, molecular, and transport biology of Malpighian tubules.. Annual Review of Entomology.

[9] Sözen MA, Armstrong JD, Yang MY, Kaiser K, Dow JAT (1997). Functional domains are specified to single-cell resolution in a *Drosophila* epithelium.. Proceedings of the National Academy of Sciences of the United States of America.

[10] Dow JAT, Davies SA (2003). Integrative physiology and functional genomics of epithelial function in a genetic model organism.. Physiological Reviews.

[11] Dube K, McDonald DG, O'Donnell MJ (2000). Calcium transport by isolated anterior and posterior Malpighian tubules of Drosophila melanogaster: roles of sequestration and secretion.. J Insect Physiol.

[12] Wang J, Kean L, Yang J, Allan AK, Davies SA (2004). Function-informed transcriptome analysis of *Drosophila* renal tubule.. Genome Biology.

[13] Dow JAT (2009). Insights into the Malpighian tubule from functional genomics.. J exp Biol.

[14] Dow JAT, Romero MF (2010). Drosophila provides rapid modeling of renal development, function, and disease.. Am J Physiol Renal Physiol.

[15] Singh SR, Hou SX (2009). Multipotent stem cells in the Malpighian tubules of adult *Drosophila melanogaster*.. Journal of Experimental Biology.

[16] Singh SR, Liu W, Hou SX (2007). The adult *Drosophila* Malpighian tubules are maintained by multipotent stem cells.. Cell Stem Cell.

[17] Singh SR, Hou SX (2008). Lessons learned about adult kidney stem cells from the malpighian tubules of Drosophila.. J Am Soc Nephrol.

[18] Zeng X, Singh SR, Hou D, Hou SX (2010). Tumor suppressors Sav/Scrib and oncogene Ras regulate stem-cell transformation in adult Drosophila malpighian tubules.. J Cell Physiol.

[19] Chen YH, Liu HP, Chen HY, Tsai FJ, Chang CH (2011). Ethylene glycol induces calcium oxalate crystal deposition in Malpighian tubules: a Drosophila model for nephrolithiasis/urolithiasis.. Kidney Int.

[20] Kamleh MA, Hobani Y, Dow JAT, Watson DG (2008). Metabolomic profiling of *Drosophila* using liquid chromatography Fourier transform mass spectrometry.. FEBS Lett.

[21] Kaufmann N, Mathai JC, Hill WG, Dow JAT, Zeidel ML (2005). Developmental Expression and Biophysical Characterization of a Drosophila melanogaster Aquaporin.. American Journal of Physiology Cell Physiology.

[22] Drake LL, Boudko DY, Marinotti O, Carpenter VK, Dawe AL (2010). The Aquaporin gene family of the yellow fever mosquito, Aedes aegypti.. PLoS One.

[23] Hofmann T, Chubanov V, Chen X, Dietz AS, Gudermann T (2010). Drosophila TRPM channel is essential for the control of extracellular magnesium levels.. PLoS One.

[24] Stergiopoulos K, Cabrero P, Davies SA, Dow JAT (2008). Salty dog, an SLC5 symporter, modulates Drosophila response to salt stress..

[25] Overend G, Cabrero P, Guo AX, Sebastian S, Cundall M (2011). The receptor guanylate cyclase Gyc76C and a peptide ligand, NPLP1-VQQ, modulate the innate immune IMD pathway in response to salt stress..

[26] Terhzaz S, Cabrero P, Chintapalli VR, Davies SA, Dow JAT (2010). Mislocalization of mitochondria and compromised renal function and oxidative stress resistance in *Drosophila SesB* mutants.. Physiol Genomics.

[27] Terhzaz S, Finlayson AJ, Stirrat L, Yang J, Tricoire H (2010). Cell-specific inositol 1,4,5 trisphosphate 3-kinase mediates epithelial cell apoptosis in response to oxidative stress in Drosophila.. Cell Signal.

[28] Soderberg JA, Birse RT, Nassel DR (2011). Insulin production and signaling in renal tubules of *Drosophila* is under control of tachykinin-related peptide and regulates stress resistance.. PLoS One.

[29] McGettigan J, McLennan RK, Broderick KE, Kean L, Allan AK (2005). Insect renal tubules constitute a cell-autonomous immune system that protects the organism against bacterial infection.. Insect Biochem Mol Biol.

[30] Torrie LS, Radford JC, Southall TD, Kean L, Dinsmore AJ (2004). Resolution of the insect ouabain paradox.. Proceedings of the National Academy of Sciences of the United States of America.

[31] Yang J, McCart C, Woods DJ, Terhzaz S, Greenwood KG (2007). A Drosophila systems approach to xenobiotic metabolism.. Physiol Genomics.

[32] Chahine S, O'Donnell MJ (2009). Physiological and molecular characterization of methotrexate transport by Malpighian tubules of adult Drosophila melanogaster.. J Insect Physiol.

[33] Chahine S, O'Donnell MJ (2010). Effects of acute or chronic exposure to dietary organic anions on secretion of methotrexate and salicylate by Malpighian tubules of Drosophila melanogaster larvae.. Arch Insect Biochem Physiol.

[34] Chahine S, O'Donnell MJ (2011). Interactions between detoxification mechanisms and excretion in Malpighian tubules of Drosophila melanogaster.. J Exp Biol.

[35] Coast GM (2009). Neuroendocrine control of ionic homeostasis in blood-sucking insects.. Journal of Experimental Biology.

[36] Coast GM, Garside CS (2005). Neuropeptide control of fluid balance in insects.. Ann N Y Acad Sci.

[37] Secca T, Sciaccaluga M, Marra A, Barberini L, Bicchierai MC (2011). Biochemical activity and multiple locations of particulate guanylate cyclase in Rhyacophila dorsalis acutidens (Insecta: Trichoptera) provide insights into the cGMP signalling pathway in Malpighian tubules.. J Insect Physiol.

[38] Chintapalli VR, Wang J, Dow JAT (2007). Using FlyAtlas to identify better *Drosophila* models of human disease.. Nature Genetics.

[39] Wheat CW, Vogel H, Wittstock U, Braby MF, Underwood D (2007). The genetic basis of a plant-insect coevolutionary key innovation.. Proc Natl Acad Sci U S A.

[40] Iliadi KG, Avivi A, Iliadi NN, Knight D, Korol AB (2008). nemy encodes a cytochrome b561 that is required for Drosophila learning and memory.. Proc Natl Acad Sci U S A.

[41] Marquez J, de la Oliva AR, Mates JM, Segura JA, Alonso FJ (2006). Glutaminase: a multifaceted protein not only involved in generating glutamate.. Neurochem Int.

[42] Chapman RF (1982). The insects: structure and function..

[43] Caldwell GM, Kakuk LE, Griesinger IB, Simpson SA, Nowak NJ (1999). Bestrophin gene mutations in patients with Best vitelliform macular dystrophy.. Genomics.

[44] Marmorstein AD, Marmorstein LY, Rayborn M, Wang X, Hollyfield JG (2000). Bestrophin, the product of the Best vitelliform macular dystrophy gene (VMD2), localizes to the basolateral plasma membrane of the retinal pigment epithelium.. Proc Natl Acad Sci U S A.

[45] Aldehni F, Spitzner M, Martins JR, Barro-Soria R, Schreiber R (2009). Bestrophin 1 promotes epithelial-to-mesenchymal transition of renal collecting duct cells.. J Am Soc Nephrol.

[46] Pifferi S, Pascarella G, Boccaccio A, Mazzatenta A, Gustincich S (2006). Bestrophin-2 is a candidate calcium-activated chloride channel involved in olfactory transduction.. Proc Natl Acad Sci U S A.

[47] Chien LT, Hartzell HC (2007). Drosophila bestrophin-1 chloride current is dually regulated by calcium and cell volume.. J Gen Physiol.

[48] O'Donnell MJ, Dow JAT, Huesmann GR, Tublitz NJ, Maddrell SHP (1996). Separate control of anion and cation transport in malpighian tubules of *Drosophila melanogaster*.. Journal of Experimental Biology.

[49] O'Donnell MJ, Rheault MR, Davies SA, Rosay P, Harvey BJ (1998). Hormonally controlled chloride movement across *Drosophila* tubules is via ion channels in stellate cells.. American Journal of Physiology.

[50] Wessing A, Zierold K (1999). The formation of type I concretions in *Drosophila* Malpighian tubules studied by electron microscopy and X-ray microanalysis.. Journal of Insect Physiology.

[51] Southall TD, Terhzaz S, Cabrero P, Chintapalli VR, Evans JM (2006). Novel subcellular locations and functions for secretory pathway Ca^2+^/Mn^2+^-ATPases.. Physiol Genomics.

[52] Reim I, Frasch M (2005). The Dorsocross T-box genes are key components of the regulatory network controlling early cardiogenesis in Drosophila.. Development.

[53] Hatton-Ellis E, Ainsworth C, Sushama Y, Wan S, VijayRaghavan K (2007). Genetic regulation of patterned tubular branching in *Drosophila*.. Proc Natl Acad Sci U S A.

[54] Metaxakis A, Oehler S, Klinakis A, Savakis C (2005). Minos as a genetic and genomic tool in Drosophila melanogaster.. Genetics.

[55] Eden E, Navon R, Steinfeld I, Lipson D, Yakhini Z (2009). GOrilla: a tool for discovery and visualization of enriched GO terms in ranked gene lists.. BMC Bioinformatics.

[56] Fujii S, Amrein H (2002). Genes expressed in the Drosophila head reveal a role for fat cells in sex-specific physiology.. EMBO J.

[57] Wolfner MF (2003). Sex determination: sex on the brain?. Curr Biol.

[58] Yapici N, Kim YJ, Ribeiro C, Dickson BJ (2008). A receptor that mediates the post-mating switch in Drosophila reproductive behaviour.. Nature.

[59] Kim YJ, Bartalska K, Audsley N, Yamanaka N, Yapici N (2010). MIPs are ancestral ligands for the sex peptide receptor.. Proc Natl Acad Sci U S A.

[60] Hewes RS, Taghert PH (2001). Neuropeptides and neuropeptide receptors in the Drosophila melanogaster genome.. Genome Res.

[61] Vanden Broeck J (2001). Neuropeptides and their precursors in the fruitfly, Drosophila melanogaster.. Peptides.

[62] Veenstra JA (2009). Peptidergic paracrine and endocrine cells in the midgut of the fruit fly maggot.. Cell Tissue Res.

[63] Feng G, Reale V, Chatwin H, Kennedy K, Venard R (2003). Functional characterization of a neuropeptide F-like receptor from Drosophila melanogaster.. Eur J Neurosci.

[64] Garczynski SF, Brown MR, Shen P, Murray TF, Crim JW (2002). Characterization of a functional neuropeptide F receptor from Drosophila melanogaster.. Peptides.

[65] Johnson EC, Bohn LM, Barak LS, Birse RT, Nassel DR (2003). Identification of Drosophila neuropeptide receptors by G protein-coupled receptors-beta-arrestin2 interactions.. J Biol Chem.

[66] Mertens I, Meeusen T, Huybrechts R, De Loof A, Schoofs L (2002). Characterization of the short neuropeptide F receptor from Drosophila melanogaster.. Biochem Biophys Res Commun.

[67] Nassel DR, Wegener C (2011). A comparative review of short and long neuropeptide F signaling in invertebrates: Any similarities to vertebrate neuropeptide Y signaling?. Peptides.

[68] Lee KS, You KH, Choo JK, Han YM, Yu K (2004). Drosophila short neuropeptide F regulates food intake and body size.. J Biol Chem.

[69] Wu Q, Wen T, Lee G, Park JH, Cai HN (2003). Developmental control of foraging and social behavior by the Drosophila neuropeptide Y-like system.. Neuron.

[70] Lee G, Bahn JH, Park JH (2006). Sex- and clock-controlled expression of the neuropeptide F gene in Drosophila.. Proc Natl Acad Sci U S A.

[71] MacPherson MR, Pollock VP, Broderick KE, Kean L, O'Connell FC (2001). Model organisms: new insights into ion channel and transporter function. L-type calcium channels regulate epithelial fluid transport in Drosophila melanogaster.. Am J Physiol Cell Physiol.

[72] Broderick KE, MacPherson MR, Regulski M, Tully T, Dow JA (2003). Interactions between epithelial nitric oxide signaling and phosphodiesterase activity in Drosophila.. Am J Physiol Cell Physiol.

[73] Wen T, Parrish CA, Xu D, Wu Q, Shen P (2005). Drosophila neuropeptide F and its receptor, NPFR1, define a signaling pathway that acutely modulates alcohol sensitivity.. Proc Natl Acad Sci U S A.

[74] Huang Y, Crim JW, Nuss AB, Brown MR (2011). Neuropeptide F and the corn earworm, Helicoverpa zea: a midgut peptide revisited.. Peptides.

[75] Kahsai L, Kapan N, Dircksen H, Winther AM, Nässel DR (2010). Metabolic stress responses in Drosophila are modulated by brain neurosecretory cells that produce multiple neuropeptides.. PLoS One.

[76] Tzou P, De Gregorio E, Lemaitre B (2002). How Drosophila combats microbial infection: a model to study innate immunity and host-pathogen interactions.. Curr Opin Microbiol.

[77] Mellroth P, Steiner H (2006). PGRP-SB1: an N-acetylmuramoyl L-alanine amidase with antibacterial activity.. Biochem Biophys Res Commun.

[78] Winterhalter WE, Fedorka KM (2009). Sex-specific variation in the emphasis, inducibility and timing of the post-mating immune response in Drosophila melanogaster.. Proc Biol Sci.

[79] Rosay P, Davies SA, Yu Y, Sozen MA, Kaiser K (1997). Cell-type specific calcium signalling in a *Drosophila* epithelium.. Journal of Cell Science 110 ( Pt.

[80] Dow JAT, Maddrell SHP, Görtz A, Skaer NV, Brogan S (1994). The Malpighian tubules of *Drosophila melanogaster:* a novel phenotype for studies of fluid secretion and its control.. J exp Biol.

[81] Evans JM, Allan AK, Davies SA, Dow JAT (2005). Sulphonylurea sensitivity and enriched expression implicate inward rectifier K^+^ channels in *Drosophila melanogaster* renal function.. J expBiol.

[82] Allan AK, Du J, Davies SA, Dow JAT (2005). Genome-wide survey of V-ATPase genes in Drosophila reveals a conserved renal phenotype for lethal alleles.. Physiol Genomics.

[83] Coast GM, Webster SG, Schegg KM, Tobe SS, Schooley DA (2001). The Drosophila melanogaster homologue of an insect calcitonin-like diuretic peptide stimulates V-ATPase activity in fruit fly Malpighian tubules.. J Exp Biol.

[84] Terhzaz S, O'Connell FC, Pollock VP, Kean L, Davies SA (1999). Isolation and characterization of a leucokinin-like peptide of Drosophila melanogaster.. Journal of Experimental Biology.

[85] Davies SA, Huesmann GR, Maddrell SHP, O'Donnell MJ, Skaer NJ (1995). CAP2b, a cardioacceleratory peptide, is present in *Drosophila* and stimulates tubule fluid secretion via cGMP.. American Journal of Physiology.

[86] Kamleh MA, Hobani Y, Dow JAT, Zheng L, Watson DG (2009). Towards a platform for the metabonomic profiling of different strains of *Drosophila melanogaster* using liquid chromatography Fourier transform mass spectrometry..

[87] Drago I, Giacomello M, Pizzo P, Pozzan T (2008). Calcium dynamics in the peroxisomal lumen of living cells.. J Biol Chem.

[88] Rosay P, Davies SA, Yu Y, Sozen A, Kaiser K (1997). Cell-type specific calcium signalling in a Drosophila epithelium.. J Cell Sci 110 ( Pt.

[89] Southall TD, Terhzaz S, Cabrero P, Chintapalli VR, Evans JM (2006). Novel subcellular locations and functions for secretory pathway Ca2+/Mn2+-ATPases.. Physiol Genomics.

[90] Button D, Eidsath A (1996). Aequorin targeted to the endoplasmic reticulum reveals heterogeneity in luminal Ca++ concentration and reports agonist- or IP3-induced release of Ca++.. Mol Biol Cell.

[91] Dow JA, Maddrell SH, Görtz A, Skaer NJ, Brogan S (1994). The malpighian tubules of Drosophila melanogaster: a novel phenotype for studies of fluid secretion and its control.. J Exp Biol.

