# Supplementary material for: Functional Correlates of Positional and Gender-Specific Renal Asymmetry in Drosophila
Source: PLoS One. 2012 Apr 4;7(4):e32577. doi: 10.1371/journal.pone.0032577 (PMC3319558; doi:10.1371/journal.pone.0032577)
Supplement: Supporting information S1 — The combined supplementary information file comprises Figures S1 to S4 and Tables S1 to S4. (DOCX) [file pone.0032577.s001.docx]

### Supporting information S1

### Supplementary Figures

**Fig. S1**

**Fig. S1. Analysis of correlation of qPCR and Microarray fold changes (FCs).** Total of 13 genes including 4 anterior, 3 posterior, 3 female, 3 male were selected for qPCR validation. The data shows the agreement (Pearson r = 0.7, 95% confidence interval, P = 0.007) between qPCR and Microarray FCs. Genes with expression FCs exceeding 50 found to be highly specific to one of the two comparison groups; for example, *CG14963* shows expression in only anterior tubules. Very high FC values were plotted as 50 for both qPCR and microarray technologies so that all points could appear on the same graph. Rpl32 was used as the reference control in the qPCR experiments. Both qPCR and Microarray (X/Y) fold changes for A = anterior; P = posterior; F = female; M = male are shown in the graph; so for example, “*nemy* P:6/4” means that the *nemy* gene was found to be posterior-enriched by both qPCR (6-fold) and microarray (4 fold).

**Fig. S2**


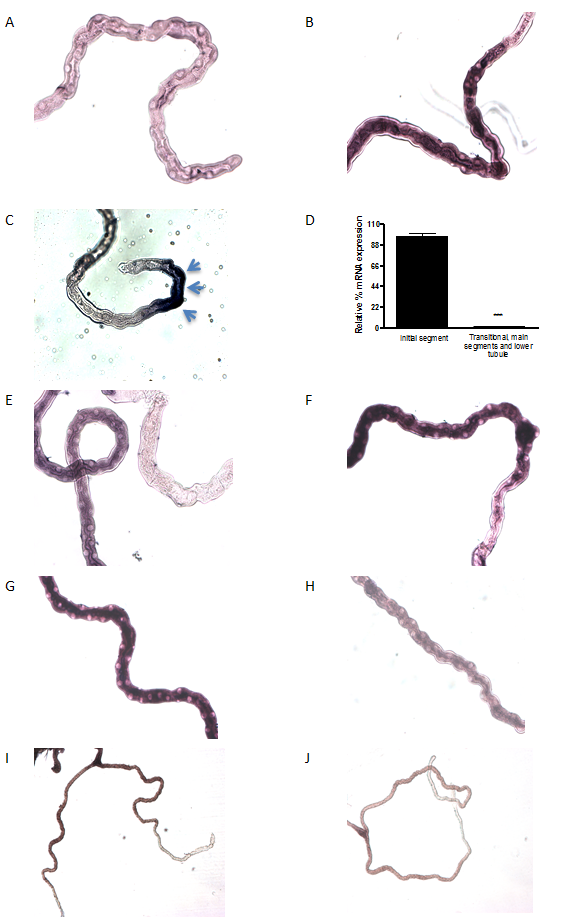


**Fig. S2. Validation of asymmetric expression patterns by *in situ* hybridization.** The anti-sense mRNA *in situ* hybridization of *CG14963* in posterior (A), and anterior (B) tubules. Anterior tubules show greater anti-sense hybridization signal than posterior. (C) *In situ* hybridization of right-hand (anterior) tubule with *Bestrophin2* anti-sense probe. Initial segment is arrowed. No staining was observed with sense probes, or in left tubules. (D) qPCR showing initial segment specific expression of *Best2* in adult anterior Malpighian tubules. **Sexually dimorphic gene expression patterns**: Gender-specific transcription is also confirmed by anti-sense mRNA *in situ* hybridization. *In situ* hybridization of *CG3599* is lower in male (E) than female (F) tubule main segments; *CG9657* is greater in males (G), than females (H) tubule main segments; *doublesex-RA* is greater in male (I) than female (J) anterior tubules. qPCR confirmation of gender specific enrichments of these genes along with others is shown in Supplementary Table 1.

**Fig. S3**


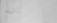


Best2-Aeq 110kD

Cytosol

Peroxisomes

**Fig. S3. Best2 is peroxisomal.** Western blot of Best2-Aeq protein from the purified peroxisomal fractions along with a negative control protein blot probing the rest of the cytosolic protein fraction for Best2-Aeq. A peroxisomal fraction was obtained from whole flies expressing the Best2-aequorin translational fusion, using a peroxisomal purification kit (Pierce), according to the manufacturer’s instructions. Peroxisomal enrichment was monitored as catalase enrichment.

**Fig. S4**

**B**

**A**

**Fig. S4.** Gene expression patterns of *Neuropeptide F* (*NPF*) in the adult heads and bodies. (A) qPCR analysis of *NPF* in females and males; heads and bodies separately. The data are presented as the % expression relative to females. *NPF* is more enriched in the heads than bodies with mean difference 74.0 ± 11.8 % (P < 0.0001); and is consistently enriched more in the males than females both in heads and bodies with mean difference of 44.8 ± 19.3 % (*, P=0.037) and 22.9 ± 6.7 (**, P = 0.004) respectively. (B) FlyAtlas gene expression pattern across tissues, confirms *NPF* expression in brain (but not in ventral nerve cord) and midgut both in adult and larval stages.

### Supporting Tables

*Table S1.* Probesets with significant expression differences (at FDR of P<0.05) between right (anterior) and left (posterior) tubules.

| Probeset ID | Gene Symbol | Gene Title | p-value(Tissue) | Fold-Change(A vs. P) |
| --- | --- | --- | --- | --- |
| 1638974_at | *CG14963* | *CG14963* | 1.93E-19 | 1940.88 |
| 1640881_at | *CG16762* | *CG16762* | 1.71E-10 | 239.659 |
| 1634031_at | *Best2* | *Bestrophin 2* | 1.40E-10 | 63.5515 |
| 1631218_at | *Doc1* | *Dorsocross* | 5.03E-12 | 52.3512 |
| 1627384_at | *CG6225* | *CG6225* | 2.84E-08 | 18.503 |
| 1629459_at | *Doc3* | *Dorsocross3* | 3.78E-12 | 16.6997 |
| 1635591_at | *NijA* | *ninjurin A* | 2.74E-10 | 15.2286 |
| 1625266_at | *Ag5r* | *antigen 5-related* | 1.05E-05 | 12.4774 |
| 1628302_at | *CG13748* | *CG13748* | 1.44E-09 | 10.6488 |
| 1635398_at | *CG10587* | *CG10587* | 2.84E-08 | 6.21122 |
| 1629948_at | *CG5194* | *CG5194* | 1.05E-06 | 5.23835 |
| 1641413_s_at | *CG3074* | *CG3074* | 1.05E-07 | 5.08742 |
| 1640854_at | *CG31248* | *CG31248* | 3.74E-09 | 5.0542 |
| 1624660_at | *Ptp10D* | *Protein tyrosine phosphatase 10D* | 4.03E-09 | 4.72262 |
| 1630088_at | *CG16743* | *CG16743* | 1.57E-08 | 4.60355 |
| 1636474_at | *CG12602* | *CG12602* | 2.03E-09 | 4.40782 |
| 1632918_at | *CG18746* | *CG18746* | 2.75E-06 | 4.26911 |
| 1636374_at | *CAH1* | *Carbonic anhydrase 1* | 1.23E-06 | 4.25813 |
| 1629916_at | *debcl* | *deborg* | 3.66E-09 | 4.16467 |
| 1638077_at | *CG7634* | *CG7634* | 3.42E-07 | 4.04019 |
| 1633341_s_at | *dac* | *Dachshund* | 1.83E-05 | 3.95692 |
| 1624577_at | *Ace* | *acetylcholine esterase* | 1.10E-08 | 3.51503 |
| 1624181_at | *out* | *outsiders* | 7.10E-05 | 3.43072 |
| 1634828_at | *pgant8* | *polypeptide GalNAc transferase 8* | 1.89E-08 | 3.42025 |
| 1626074_at | *CG34454* | *---* | 1.48E-05 | 3.34883 |
| 1631524_a_at | *hth* | *dorsotonals* | 3.30E-08 | 3.26943 |
| 1636265_s_at | *Btk29A* | *Btk family kinase at 29A* | 5.30E-07 | 3.14005 |
| 1623853_at | *betaggt-I* | *type I Geranylgeranyl Transferase* | 1.30E-06 | 3.06347 |
| 1639320_a_at | *Ddc* | *Dopa decarboxylase* | 4.01E-06 | 3.03603 |
| 1637605_s_at | *CG1146* | *CG1146* | 7.29E-07 | 2.73723 |
| 1634818_s_at | *CG42314* | *CG42314* | 1.86E-07 | 2.69998 |
| 1631608_at | *CG6954* | *CG6954* | 1.04E-06 | 2.69589 |
| 1633582_at | *Ih* | *putative Ih-channel* | 1.63E-06 | 2.6158 |
| 1631568_a_at | *sdt* | *CG12657* | 5.00E-09 | 2.61408 |
| 1639624_at | *CG6688* | *CG6688* | 8.18E-06 | 2.5747 |
| 1626081_at | *CG8837* | *CG8837* | 7.52E-07 | 2.4715 |
| 1627961_a_at | *CG18507* | *CG18507* | 1.06E-06 | 2.29762 |
| 1631274_at | *CG18095* | *gp150-like* | 2.80E-05 | 2.23849 |
| 1638312_at | *CG13189* | *CG13189* | 4.33E-07 | 2.23458 |
| 1626124_at | *CG9717* | *CG9717* | 2.33E-08 | 2.21586 |
| 1641310_at | *ko* | *knockout* | 5.96E-05 | 2.18748 |
| 1634557_at | *Rhp* | *Rhophilin* | 8.45E-07 | 2.13755 |
| 1639335_at | *unknown* | *---* | 3.00E-06 | 2.12564 |
| 1638724_at | *CG18507* | *CG18507* | 1.65E-05 | 2.09275 |
| 1640747_s_at | *CG8547* | *CG8547* | 1.94E-06 | 2.08754 |
| 1630676_at | *CG30116* | *CG30116* | 1.42E-05 | 1.88332 |
| 1639635_s_at | *CG42542* | *---* | 1.77E-06 | 1.80539 |
| 1634063_a_at | *stumps* | *heartbroken* | 6.33E-05 | 1.80312 |
| 1632650_at | *CG5867* | *CG5867* | 1.02E-07 | 1.79515 |
| 1627744_at | *CG15209* | *CG15209* | 2.38E-06 | 1.78683 |
| 1637352_a_at | *CG18812* | *CG18812* | 3.62E-06 | 1.77495 |
| 1635482_at | *CG2976* | *CG2976* | 1.73E-07 | 1.75695 |
| 1636953_a_at | *Lmpt* | *Limpet* | 0.000186 | 1.74574 |
| 1628268_at | *CG18249* | *CG18249* | 3.70E-05 | 1.71547 |
| 1625476_a_at | *CG1674* | *CG1674* | 5.93E-05 | 1.6942 |
| 1628261_at | *CG13067* | *CG13067* | 1.33E-05 | 1.68701 |
| 1630999_at | *CG7724* | *CG7724* | 2.67E-05 | 1.68625 |
| 1631526_s_at | *CG31284* | *water witch* | 5.84E-06 | 1.6782 |
| 1636423_at | *CG8012* | *CG8012* | 9.51E-05 | 1.67114 |
| 1628328_at | *GstE10* | *Glutathione S transferase E10* | 2.33E-05 | 1.65665 |
| 1625891_at | *CG17111* | *CG17111* | 0.000112 | 1.65491 |
| 1634463_at | *CG7992* | *CG7992* | 4.56E-08 | 1.65211 |
| 1624225_at | *danr* | *Distal antenna related* | 0.000108 | 1.6388 |
| 1635890_at | *capaR* | *capa receptor* | 1.16E-05 | 1.6228 |
| 1632558_at | *CG5630* | *CG5630* | 1.85E-05 | 1.61542 |
| 1624017_at | *Ahcy89E* | *AdoHcyase-like* | 0.000138 | 1.58219 |
| 1637900_at | *CG11852* | *CG11852* | 7.90E-05 | 1.57078 |
| 1625075_at | *Nep2* | *Neprilysin 2* | 8.19E-06 | 1.55144 |
| 1631573_a_at | *wun* | *wunen* | 1.82E-05 | 1.52748 |
| 1623066_at | *Irk3* | *Inwardly rectifying potassium channel 3* | 0.000135 | 1.47707 |
| 1628990_at | *Hmgcr* | *columbus* | 2.25E-06 | 1.40475 |
| 1637671_a_at | *CG14767* | *CG14767* | 9.32E-05 | 1.38802 |
| 1636501_at | *Muc11A* | *Mucin 11A* | 6.61E-10 | 1.28682 |
| 1625814_at | *CG8230* | *CG8230* | 0.000142 | 1.24257 |
| 1638809_at | *CG34284* | *---* | 0.00015 | -1.20485 |
| 1631004_s_at | *su(Hw)* | *suppressor of hairy-wing* | 0.000161 | -1.2369 |
| 1641365_s_at | *multiple hits* | *---* | 9.52E-05 | -1.25591 |
| 1627761_at | *fat2* | *fat-like* | 1.28E-07 | -1.48107 |
| 1624060_at | *bab2* | *bric-a-brac* | 0.00017 | -1.5376 |
| 1626439_at | *CG15353* | *CG15353* | 0.000182 | -1.86275 |
| 1635803_s_at | *bowl* | *bowel* | 8.66E-07 | -1.93196 |
| 1634418_at | *CG33281* | *CG33281* | 1.52E-07 | -2.43081 |
| 1637123_a_at | *CG33281* | *CG33281* | 3.50E-07 | -2.72487 |
| 1627354_at | *CG11779* | *CG11779* | 0.000132 | -3.32843 |
| 1634658_a_at | *nemy* | *no extended memory* | 2.01E-05 | -3.75797 |
| 1625629_at | *alpha-Est6* | *Esterase-6* | 3.89E-08 | -3.83112 |
| 1639637_a_at | *CG3376* | *CG3376* | 5.09E-07 | -7.59369 |
| 1629738_at | *CG14957* | *CG14957* | 9.18E-07 | -9.03153 |

### Table S2. Supplementary Table 2. Probesets significantly enriched (at FDR of P<0.05, and with 2-fold change cut-off) in male or female adult tubules.

| **Probeset ID** | **Gene Symbol** | **Gene Title** | ***P*-value(F vs. M)** | **Fold-Change (F vs. M)** |
| --- | --- | --- | --- | --- |
| 1629545_at | *Yp1* | *yolk protein 1* | 5.44661E-07 | 515.718 |
| 1631419_at | *Yp3* | *yolk protein* | 4.41882E-07 | 514.01 |
| 1623655_at | *Yp2* | *yolk protein 2* | 2.28907E-07 | 387.74 |
| 1633540_at | *CG8147* | *CG8147* | 4.96236E-07 | 39.8852 |
| 1630600_at | *Fst* | *Frost* | 2.12495E-05 | 31.4196 |
| 1637702_at | *CG34427* | *---* | 1.1743E-07 | 28.8454 |
| 1641419_at | *AttC* | *attacin* | 0.000672278 | 25.1226 |
| 1633820_at | *Fad2* | *Fad2* | 0.000354706 | 22.46 |
| 1623776_s_at | *dsx* | *doublesex* | 1.42375E-10 | 19.3021 |
| 1627613_at | *Mtk* | *Metchnikowin* | 7.05154E-05 | 12.0313 |
| 1627088_at | *fit* | *female-specific independent of transformer* | 0.000312061 | 9.92567 |
| 1636490_at | *PGRP-SB1* | *PGRP-SB1* | 0.0013889 | 9.28792 |
| 1633200_at | *CG9449* | *CG9449* | 8.81902E-07 | 9.19493 |
| 1636970_at | *CG9394* | *CG9394* | 1.71784E-07 | 8.95865 |
| 1631691_at | *CG16772* | *CG16772 /// --- /// ---* | 0.000940336 | 8.36051 |
| 1635507_at | *CG30031 /// CG4269* | *CG30031 /// CG4269* | 0.000814004 | 7.64963 |
| 1640360_at | *IM2* | *Immune induced molecule 2* | 0.001966 | 7.43223 |
| 1640144_at | *CG18067* | *CG18067* | 0.000806396 | 7.31914 |
| 1627262_at | *Btd* | *CG3599* | 2.06486E-08 | 6.54484 |
| 1623068_at | *Cyp4e3* | *Cytochrome P450-4e3* | 9.24683E-06 | 6.54228 |
| 1629540_a_at | *bw* | *Plum* | 9.92022E-06 | 6.30251 |
| 1628150_a_at | *CG9449* | *CG9449* | 9.56221E-08 | 6.13289 |
| 1629732_at | *CG8791* | *CG8791* | 7.99963E-05 | 5.95411 |
| 1623643_s_at | *Idgf3* | *Imaginal disc growth factor 3* | 6.51519E-06 | 5.89748 |
| 1632882_at | *CG11671* | *CG11671* | 7.4485E-07 | 5.83668 |
| 1638991_at | *CG5506* | *CG5506 /// ---* | 7.20492E-05 | 5.30937 |
| 1633237_at | *Idgf1* | *Imaginal disc growth factor1* | 0.000216353 | 5.13182 |
| 1641327_at | *CG9416* | *CG9416* | 0.000502528 | 5.11898 |
| 1635109_at | *CG5888* | *CG5888* | 0.000778925 | 5.01207 |
| 1626301_at | *Idgf3* | *Imaginal disc growth factor 3* | 0.000502393 | 4.86595 |
| 1628398_at | *CG5853* | *CG5853* | 1.89019E-05 | 4.5902 |
| 1637958_at | *CG33510* | *CG33510* | 1.73491E-05 | 4.50143 |
| 1634731_at | *Cyp4p3* | *Cyp4p3* | 4.90471E-06 | 4.42895 |
| 1623928_at | *CG5910* | *CG5910* | 0.000323557 | 4.4027 |
| 1632082_at | *Ndg* | *Nidogen-like* | 1.72415E-07 | 4.10305 |
| 1635688_at | *CG15210* | *CG15210* | 4.17047E-05 | 4.07384 |
| 1625603_at | *CG13604* | *CG13604* | 9.2886E-07 | 3.99007 |
| 1625149_at | *CG7460* | *CG7460* | 0.00149366 | 3.90624 |
| 1629776_a_at | *CG6643* | *CG6643* | 0.00013162 | 3.89415 |
| 1638811_at | *Sug* | *Sugar-baby* | 8.81831E-11 | 3.81651 |
| 1639637_a_at | *CG3376* | *CG3376* | 0.000042076 | 3.81612 |
| 1640950_at | *CG15739* | *CG15739* | 0.000858702 | 3.7731 |
| 1633857_at | *CG13659* | *CG13659* | 9.41942E-07 | 3.66098 |
| 1624471_s_at | *Act42A* | *actin* | 0.000185995 | 3.54419 |
| 1634552_at | *TepIV* | *Thiolester containing protein IV* | 0.000131701 | 3.52566 |
| 1629007_at | *CG8952* | *CG8952* | 5.18993E-06 | 3.43354 |
| 1638849_a_at | *CG2875* | *CG2875* | 1.38757E-06 | 3.28758 |
| 1623777_s_at | *CG1358* | *CG1358* | 6.81637E-06 | 3.27266 |
| 1639528_at | *NAAT1* | *CG3252* | 8.63122E-05 | 3.26974 |
| 1626028_at | *CG4783* | *CG4783* | 3.32725E-08 | 3.25795 |
| 1632030_at | *CG31454* | *CG31454* | 6.32093E-07 | 3.23855 |
| 1631007_at | *RnrL* | *ribonucleoside-diphosphate reductase large subunit* | 1.04115E-05 | 3.18834 |
| 1630642_at | *Pvf2* | *VEGF-related factor 2* | 0.00146108 | 3.02848 |
| 1627395_a_at | *CG34123* | *CG34123* | 0.000201865 | 3.0119 |
| 1630584_at | *CG10657* | *retinaldehyde-binding related protein* | 3.42617E-06 | 2.97672 |
| 1641333_s_at | *prominin-like* | *prominin-like protein* | 0.00168041 | 2.97499 |
| 1639498_a_at | *ap* | *Xasta* | 5.88565E-05 | 2.96082 |
| 1640262_at | *CG34123* | *CG34123* | 0.00219904 | 2.92378 |
| 1632179_at | *Sptr* | *sepiapterin reductase* | 1.43133E-09 | 2.91298 |
| 1636736_s_at | *CG31274 /// MESK4* | *CG31274 /// Misexpression suppressor of KSR 4* | 0.000602578 | 2.88136 |
| 1623761_at | *CG15347* | *CG15347* | 4.19536E-05 | 2.86849 |
| 1623258_at | *CG12493* | *CG12493* | 5.28348E-09 | 2.84846 |
| 1634445_at | *CG32750* | *CG32750* | 5.03768E-05 | 2.82858 |
| 1636775_at | *CG18622* | *CG18622* | 0.00026987 | 2.81154 |
| 1626444_at | *Fib* | *Fibrillarin* | 0.000842281 | 2.80241 |
| 1632011_at | *CG31274 /// MESK4* | *CG31274 /// Misexpression suppressor of KSR 4* | 7.49654E-05 | 2.71969 |
| 1625688_at | *CG6293* | *CG6293* | 3.60648E-06 | 2.71774 |
| 1633592_a_at | *CREG* | *cellular repressor of E1A-stimulated genes* | 0.00206409 | 2.70966 |
| 1626456_at | *l(1)G0320* | *lethal (1) G0320* | 0.000136484 | 2.68851 |
| 1631969_at | *CG15221* | *CG15221* | 2.35976E-05 | 2.67338 |
| 1634860_at | *CG34123* | *CG34123* | 0.00160922 | 2.66005 |
| 1627111_at | *NHP2* | *NHP2* | 4.20537E-05 | 2.65988 |
| 1631474_s_at | *Gpo-1* | *mitochondrial GPD* | 7.68138E-06 | 2.63733 |
| 1639554_at | *---* | *---* | 0.00109353 | 2.56603 |
| 1636040_at | *CG10527* | *CG10527* | 0.00125299 | 2.56302 |
| 1630085_s_at | *Peritrophin-A* | *peritrophin A* | 0.00125571 | 2.55504 |
| 1632348_at | *CG9452* | *CG9452* | 0.000463497 | 2.53594 |
| 1637430_s_at | *CG1572* | *CG1572* | 0.000375067 | 2.50433 |
| 1627000_s_at | *CG6231* | *CG6231* | 0.000139392 | 2.50372 |
| 1640223_a_at | *trol* | *Perlecan* | 0.000282998 | 2.50046 |
| 1633923_at | *CG1749* | *CG1749* | 4.51059E-08 | 2.49911 |
| 1626061_at | *CG34123* | *CG34123* | 9.29524E-05 | 2.47864 |
| 1630038_at | *pyd3* | *pyd3* | 5.34508E-07 | 2.46955 |
| 1630528_at | *CG17751* | *CG17751* | 0.000180961 | 2.40412 |
| 1630375_at | *Mec2* | *Mec2* | 0.000012467 | 2.39898 |
| 1624103_at | *CG7246* | *CG7246* | 0.000347238 | 2.38379 |
| 1627354_at | *CG11779 /// CG5835* | *CG11779 /// CG5835 /// --- /// CG11779 /// CG11779 /// CG11779 /// CG11779 /// C* | 0.00201067 | 2.37527 |
| 1632076_a_at | *CG6933* | *CG6933* | 0.000206104 | 2.35196 |
| 1637785_at | *L* | *Lobe* | 1.56782E-07 | 2.35062 |
| 1637703_a_at | *Socs36E* | *Suppressor of cytokine signaling at 36E* | 0.00173824 | 2.29335 |
| 1626801_at | *CG10638* | *CG10638* | 3.74651E-05 | 2.29 |
| 1631394_at | *CG31324* | *CG31324* | 0.00202019 | 2.29 |
| 1628584_at | *Cyp305a1* | *Cyp305a1* | 0.00229606 | 2.25752 |
| 1634207_at | *CG16799* | *CG16799* | 8.27143E-06 | 2.25185 |
| 1629288_s_at | *CG12608 /// CG9123* | *CG12608 /// CG9123* | 2.29487E-05 | 2.24829 |
| 1632381_at | *dome* | *verstopft* | 5.05386E-05 | 2.22391 |
| 1626236_at | *CG10343* | *CG10343* | 0.000384237 | 2.22174 |
| 1627152_at | *CG12918* | *CG12918* | 0.000644376 | 2.21233 |
| 1635052_a_at | *CG15747* | *CG15747* | 3.77197E-07 | 2.2108 |
| 1638226_at | *CG10562* | *CG10562* | 4.92716E-05 | 2.20703 |
| 1626196_at | *CaBP1* | *CaBP1* | 0.00230401 | 2.17103 |
| 1627032_a_at | *CG8611* | *CG8611* | 0.00218167 | 2.16858 |
| 1627617_at | *Best1* | *Bestrophin 1* | 0.00010849 | 2.15124 |
| 1630301_at | *CG12204* | *CG12204* | 5.40162E-05 | 2.11524 |
| 1637590_at | *CG42329* | *CG42329* | 0.000725191 | 2.11383 |
| 1630438_at | *CG32409* | *CG32409* | 0.000603111 | 2.0951 |
| 1637046_at | *l(2)03659* | *Hsp related* | 2.08096E-05 | 2.08365 |
| 1624450_at | *Orct* | *Organic cation transporter* | 4.28242E-06 | 2.08104 |
| 1627995_at | *Art8* | *Arginine methyltransferase 8* | 0.000282152 | 2.06675 |
| 1622895_at | *CG32075* | *CG32075* | 0.00103082 | 2.03049 |
| 1634219_a_at | *Jafrac1* | *thioredoxin peroxidase* | 1.57982E-05 | 2.02865 |
| 1641563_at | *P58IPK* | *CG8286* | 0.0011036 | 2.01418 |
| 1624010_a_at | *CG9062* | *CG9062* | 0.000248389 | 2.01407 |
| 1637353_at | *Dat* | *Arylalkylamine N-acetyltransferase-1* | 0.000205396 | -2.0016 |
| 1626472_at | *CG30340* | *CG30340* | 0.00141151 | -2.00766 |
| 1623571_a_at | *CG9328* | *CG9328* | 0.000265165 | -2.01677 |
| 1638469_s_at | *CG3857* | *CG3857* | 0.00170483 | -2.01846 |
| 1623916_at | *CG14694* | *CG14694* | 1.09108E-08 | -2.02022 |
| 1637811_s_at | *CG5273* | *CG5273* | 1.58332E-05 | -2.02291 |
| 1626164_at | *CG31380* | *CG31380* | 7.10452E-06 | -2.02689 |
| 1623212_s_at | *tamo* | *tamo* | 0.00109067 | -2.03205 |
| 1624688_at | *CG9360* | *CG9360* | 1.00376E-06 | -2.05007 |
| 1628221_at | *CG17036* | *CG17036* | 0.000363783 | -2.05559 |
| 1639566_s_at | *B4* | *B4* | 0.000433042 | -2.06427 |
| 1625428_at | *CG13707* | *CG13707* | 0.000250633 | -2.07354 |
| 1634767_at | *CG6126* | *CG6126* | 9.74391E-09 | -2.09162 |
| 1640584_at | *Cyp9f3Psi* | *Cyp9f3Psi* | 7.44856E-05 | -2.09227 |
| 1625227_at | *CG30456* | *CG30456* | 0.000135317 | -2.11645 |
| 1633794_a_at | *Pino* | *smell impaired 21F* | 0.0016798 | -2.13902 |
| 1639868_at | *CG1702* | *CG1702* | 0.00218194 | -2.1394 |
| 1631399_a_at | *CG15771* | *CG15771* | 0.00233119 | -2.1514 |
| 1639892_at | *Sodh-1* | *Sorbitol dehydrogenase like* | 6.29175E-05 | -2.15781 |
| 1623666_at | *CG10516* | *CG10516* | 6.95463E-06 | -2.17025 |
| 1628915_s_at | *Exn* | *CG3799* | 6.30711E-05 | -2.17189 |
| 1624405_a_at | *CG7059* | *CG7059* | 4.10966E-05 | -2.18457 |
| 1628013_at | *CG3597* | *CG3597* | 5.11685E-06 | -2.18528 |
| 1627651_a_at | *loco* | *locomotion defects* | 0.000282154 | -2.18794 |
| 1634411_at | *CG6506* | *CG6506* | 0.000572629 | -2.18972 |
| 1639539_at | *Cyp4e1* | *Cytochrome P450-4e1* | 1.07834E-05 | -2.19112 |
| 1630688_at | *Hsp83* | *Enhancer of seven in absentia 2* | 0.00135464 | -2.21657 |
| 1639611_at | *CG8709* | *CG8709* | 6.61132E-05 | -2.22013 |
| 1633641_a_at | *CG15611* | *CG15611* | 0.000254041 | -2.22477 |
| 1630256_at | *lectin-24Db* | *Lectin24Db* | 8.65571E-06 | -2.24099 |
| 1628226_at | *CG8539* | *CG8539* | 2.06823E-05 | -2.24343 |
| 1629641_s_at | *---* | *---* | 0.00098638 | -2.2466 |
| 1629044_at | *trbl* | *tribbles* | 0.00176662 | -2.25629 |
| 1627709_at | *CG30371* | *CG30371* | 5.57245E-05 | -2.25994 |
| 1637577_at | *Zip3* | *Zinc/iron regulated transporter-related protein 3* | 0.00138155 | -2.27122 |
| 1624503_at | *Rcd2* | *Reduction in Cnn Dots 2* | 0.00230797 | -2.27789 |
| 1634978_at | *CG13117* | *CG13117* | 1.14357E-05 | -2.30077 |
| 1638751_a_at | *CG4004* | *CG4004* | 0.00015164 | -2.30348 |
| 1635938_s_at | *Ork1* | *Open rectifier K[+] channel 1* | 0.000353521 | -2.31259 |
| 1626086_at | *CG10621* | *selenocysteine methyltransferase* | 1.82642E-05 | -2.32386 |
| 1624122_a_at | *Rapgap1* | *Rapgap1* | 8.34986E-06 | -2.33699 |
| 1636816_s_at | *CG33090* | *CG33090* | 5.40416E-06 | -2.33734 |
| 1632517_at | *CG7966* | *CG7966* | 5.20131E-05 | -2.33785 |
| 1635639_a_at | *Eip74EF* | *ecdysone inducible protein* | 0.00218296 | -2.3434 |
| 1633563_at | *CG1637* | *CG1637* | 0.00183446 | -2.36514 |
| 1632807_at | *Dhap-at* | *Glycerol 3-phosphate acyltransferase* | 0.000414297 | -2.38926 |
| 1640109_at | *Ugt37b1* | *UDP-glycosyltransferase 37b1* | 0.000540237 | -2.39306 |
| 1637056_s_at | *CG11200* | *CG11200* | 3.88011E-05 | -2.41486 |
| 1632321_a_at | *mthl7* | *Mth-like 7* | 8.96395E-05 | -2.43096 |
| 1636674_at | *CG11407* | *CG11407* | 1.35692E-05 | -2.44658 |
| 1633881_at | *CG42347* | *MLCK-like* | 0.00010874 | -2.4701 |
| 1622946_at | *CG6908* | *CG6908* | 1.86769E-06 | -2.47661 |
| 1626769_s_at | *CG1553* | *CG1553* | 0.000404024 | -2.49591 |
| 1625697_at | *Cyp6g1* | *CYP6-like* | 7.82945E-06 | -2.50567 |
| 1638487_at | *---* | *---* | 0.000469347 | -2.51921 |
| 1626074_at | *---* | *---* | 0.000181666 | -2.54092 |
| 1626237_at | *CG12734* | *CG12734* | 0.00231308 | -2.5511 |
| 1641587_at | *CG14777 /// CG14778* | *CG14777 /// CG14778* | 0.000921228 | -2.55156 |
| 1636764_at | *CG31075* | *CG31075* | 0.000196566 | -2.553 |
| 1624839_at | *h* | *hairy* | 0.00116591 | -2.56198 |
| 1640754_at | *bmm* | *CG5295* | 4.55505E-05 | -2.56358 |
| 1640002_at | *CG4586* | *CG4586* | 0.00186308 | -2.57663 |
| 1624144_at | *CG13566* | *CG13566* | 0.000144499 | -2.58356 |
| 1632500_at | *CG10226* | *CG10226* | 3.69138E-05 | -2.62587 |
| 1633227_s_at | *CG14945* | *CG14945* | 6.81915E-07 | -2.63239 |
| 1630145_s_at | *Tsp42Ea* | *tetraspanin 42E* | 2.15383E-08 | -2.64487 |
| 1625629_at | *alpha-Est6* | *Esterase-6* | 1.53447E-06 | -2.65428 |
| 1632574_at | *CG6574* | *CG6574* | 1.11849E-07 | -2.65627 |
| 1636495_at | *---* | *---* | 0.00101347 | -2.67033 |
| 1629981_at | *LamC* | *lamin C* | 0.00237416 | -2.6794 |
| 1634337_s_at | *Sxl* | *sex-lethal* | 0.000087907 | -2.75119 |
| 1628446_at | *MtnC* | *Metallothionein C* | 0.000435498 | -2.75129 |
| 1632812_at | *Eo* | *Ecdysone oxidase* | 0.000210826 | -2.76171 |
| 1630255_at | *CG31676* | *CG31676* | 0.00021998 | -2.82012 |
| 1624797_at | *CG1637* | *CG1637* | 7.74702E-07 | -2.91535 |
| 1638742_at | *Cpr67Fb* | *CG18348* | 0.00166106 | -2.95078 |
| 1625050_s_at | *---* | *---* | 0.000534488 | -2.97957 |
| 1627242_at | *l(2)efl* | *lethal (2) essential for life* | 1.28252E-06 | -3.04131 |
| 1640714_at | *CG1979* | *CG1979* | 0.000185991 | -3.06563 |
| 1639110_at | *CG4484* | *CG4484* | 9.07873E-05 | -3.07478 |
| 1627129_at | *---* | *---* | 0.00015097 | -3.0924 |
| 1627741_at | *CG13086* | *CG13086* | 5.25032E-06 | -3.14415 |
| 1632359_at | *Takl2* | *Tak1-like 2* | 0.000394014 | -3.20374 |
| 1629806_a_at | *CG32103* | *CG32103* | 0.00111745 | -3.23466 |
| 1623446_a_at | *CG14245 /// CG14246* | *CG14245 /// CG14246* | 4.54728E-05 | -3.28527 |
| 1625897_s_at | *tal-1A /// tal-2A /// tal-3A /// tal-AA* | *CG42384 /// CG42385 /// CG42386 /// CG42387* | 0.000135575 | -3.35851 |
| 1635840_s_at | *msl-2* | *male specifc lethal* | 5.67712E-10 | -3.44935 |
| 1639069_at | *Cyp12d1-d* | *Cyp12d1-d* | 5.724E-07 | -3.49125 |
| 1627667_at | *CG16753* | *CG16753* | 6.78626E-08 | -3.52244 |
| 1635412_at | *CG7422* | *CG7422* | 0.000300579 | -3.57687 |
| 1639737_at | *CG34330* | *---* | 0.00070055 | -4.02971 |
| 1627270_at | *CG42240* | *CG42240* | 0.000409828 | -4.09964 |
| 1637388_at | *CG13511* | *CG13511* | 0.000160266 | -4.11651 |
| 1626416_a_at | *l(2)efl* | *lethal (2) essential for life* | 8.99328E-07 | -4.34113 |
| 1625795_a_at | *CG3394* | *CG3394* | 8.62184E-08 | -4.3708 |
| 1637804_at | *CG18673* | *CG18673* | 8.05529E-05 | -4.68503 |
| 1635282_at | *CG7142* | *CG7142* | 0.000956166 | -4.74013 |
| 1631407_a_at | *APC7* | *Anaphase Promoting Complex 7* | 3.23296E-06 | -4.75808 |
| 1631628_s_at | *CG12896 /// Prx2540-1 /// Prx2540-2* | *AOP2-related /// peroxiredoxin 2540 /// Peroxiredoxin 2540* | 6.17706E-06 | -4.85354 |
| 1636688_at | *Cyp4s3* | *Cyp4s3* | 0.000001001 | -4.86579 |
| 1633401_s_at | *Cyp12d1-d /// Cyp12d1-p* | *Cyp12d1-d /// Cyp12d1-p* | 1.01143E-08 | -5.11929 |
| 1631925_at | *Mdr65* | *P-glycoprotein65-Dm* | 1.30064E-05 | -5.40921 |
| 1628364_at | *CG7968* | *CG7968* | 2.19724E-05 | -5.68415 |
| 1627180_at | *Cyp4d14* | *Cyp4d14* | 1.46097E-09 | -6.41046 |
| 1623478_at | *SPR* | *CG16752* | 3.0994E-07 | -6.61793 |
| 1627653_at | *l(1)G0469* | *lethal (1) G0469* | 3.61357E-05 | -6.66035 |
| 1635189_at | *Drs* | *drosomycin* | 0.00192381 | -6.7587 |
| 1638816_at | *CG3884* | *CG3884* | 0.00112492 | -6.77181 |
| 1628982_at | *NPFR1* | *neuropeptide F receptor* | 6.98814E-07 | -6.90513 |
| 1632636_at | *CG2145* | *CG2145* | 3.14302E-09 | -8.61692 |
| 1638484_at | *Hsp67Bc* | *Gene 3* | 0.000271373 | -10.6377 |
| 1640799_at | *dsx* | *doublesex* | 6.94307E-13 | -10.837 |
| 1628611_at | *CG11241* | *CG11241* | 3.50671E-09 | -11.1295 |
| 1637145_at | *CG14787* | *CG14787* | 6.38554E-10 | -18.3459 |
| 1633432_at | *CG9657* | *CG9657* | 4.86936E-12 | -41.6033 |
| 1633275_at | *CG31562* | *CG31562* | 7.75546E-10 | -56.7831 |

**Table S3.** Detailed FlyAtlas tissue-specific gene expression patterns for the neuropeptides and their cognate receptors.

High to low gene expression is colour coded (green to light green). 'Head expression' is not colour coded if it's lower than brain as it presumably comes from the brain (see tissue descriptions in the FlyAtlas). Whole fly gene expression is not colour coded as it comes from the sum of all tissues. For every gene, Affymetrix mRNA signal, present call (signal present out of 4 biological replicates) and enrichment ('tissue' mRNA signal/whole fly mRNA signal) is presented. If the present call, for example is '0 of 4' it is called absent (or not expressed in that tissue). Abbreviations: TAG = thoracicoabdominal ganglion; SG = salivary gland; A = adult; MAG = male accessory glands; VSP = virgin spermatheca; MSP = mated spermatheca; L = larval. (www.flyatlas.org).

### Table S4. Primer pairs used for both qPCR and in situ hybridization.

| **Anterior:** | Redesigned for qPCR |
| --- | --- |
| DocF: 5'- TCCTCAGCAGGCGTTCGTTTTC -3' |  |
| DocR: 5'- TCAAGTGATGGCGGAGATACGG -3' |  |
| DacF: 5'- GCAGTTGAAGAAGCATCGCCTG -3' | Dac F: 5'- AGCATTTGGAGCGATTGCG -3' |
| DacR: 5'- TGAGCATCTGACTTTCGGGACG -3' | Dac R: 5'- CTGAGCCCGTGGACAGATTATC -3' |
| Best2F: 5'- CACTCTGATTCCAGGTCATCCG -3' |  |
| Best2R: 5'- TTGCCGTCGTCGTTATCGTG -3' |  |
| CG14963F: 5'- TCTGGACTGGCTTCTCCTGATTAC -3' |  |
| CG14963R: 5'- GGGCATCGTTGATTAGGTAGCG -3' |  |
| **Posterior:** |  |
| NemyF: 5'- CGTGGATGAGGCAGAGAACTTC -3' | Nemy F: 5'- AACTACGAGACGGGTTCTTCGG -3' |
| NemyR: 5'- GACTAACCAAGCAGCGTGAACTG -3' | Nemy R: 5'- CATCGGATTGTGTGGCTTTTTG -3' |
| FraF: 5'- ACAACAAACAGAAGCAGCAGGAC -3' | Fra F: 5'- GCGGGTATCAAGAGCCAAAATG -3' |
| FraR: 5'- GGCTCCAAGGTGAACGAAAGAG -3' | Fra R: 5'- TCAGTCCACGGTTCTCCTCAAC -3' |
| CG3376F: 5'- CTGAAGGAAAGAAAAAGTGGCAGC -3' |  |
| CG3376R: 5'- TCATAATGTGCGGACTCGTGG -3' |  |
| **Male:** |  |
| dsx-RA F: 5'- AGGTGGTAGGTCATCGGGAACATC -3' |  |
| dsx-RA R: 5'- ACAAATCTGTGTGAGCGGCAG -3' |  |
| CG31562 F: 5'- TTTCCAAGAGGCTGCTTTCAAC -3' |  |
| CG31562 R: 5'- CAGGTCCTTTTCTCCAACATCG -3' |  |
| CG9657 F: 5'- CACTTTCGTCGGTGGCAAATG -3' |  |
| CG9657 R: 5'- AGCACTTGGAACGGAACTGATG -3' |  |
| **Female:** |  |
| YP1 F: 5'- CGATTTCACCATTGAGCGTCTG -3' |  |
| YP1 R: 5'- CTTGTCACCATTGGGCTTGG -3' |  |
| CG3599 F: 5'- GCTCTTCAGCAGTTCGCATCAG -3' |  |
| CG3599 R: 5'- ATAGTCCGTTTGGTAGCAGGGC -3' |  |
| Dsx-RB F: 5'- AGGTGGTAGGTCATCGGGAACATC -3' |  |
| Dsx-RB R: 5'- TCGGGGCAAAGTAGTATTCGTTAC -3' |  |
